# Supplementary material for: Recruitment of frontal sensory circuits during visual discrimination
Source: Cell Rep. 2022 Jun 7;39(10):110932. doi: 10.1016/j.celrep.2022.110932 (PMC9247711; doi:10.1016/j.celrep.2022.110932)
Supplement: Document S1. Figures S1–S6 [file mmc1.pdf]

Cell Reports, Volume *39*

Supplemental information

Recruitment of frontal sensory circuits during visual discrimination

Eluned Broom, Vivian Imbriotis, Frank Sengpiel, William M. Connelly, and Adam Ranson

Supplemental information

Supplemental Figures

A

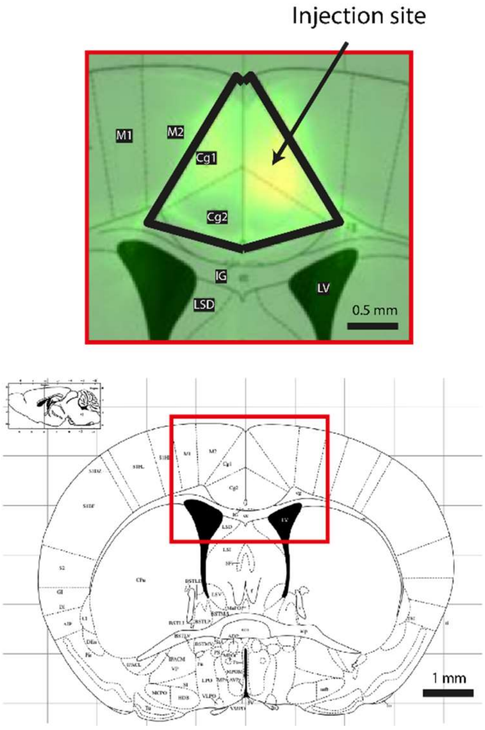

Supplemental Figure 1. The site of injection of AAV to label A24b/M2$_{axons}$. Related to Figure 1. (A) Histology from A24b injection site (labelled Cg1 in Paxinos and Franklin's the Mouse Brain in Stereotaxic Coordinates). Note, injection was unilateral, but A24b/M2 axons can be seen to project to contralateral A24b/M2.

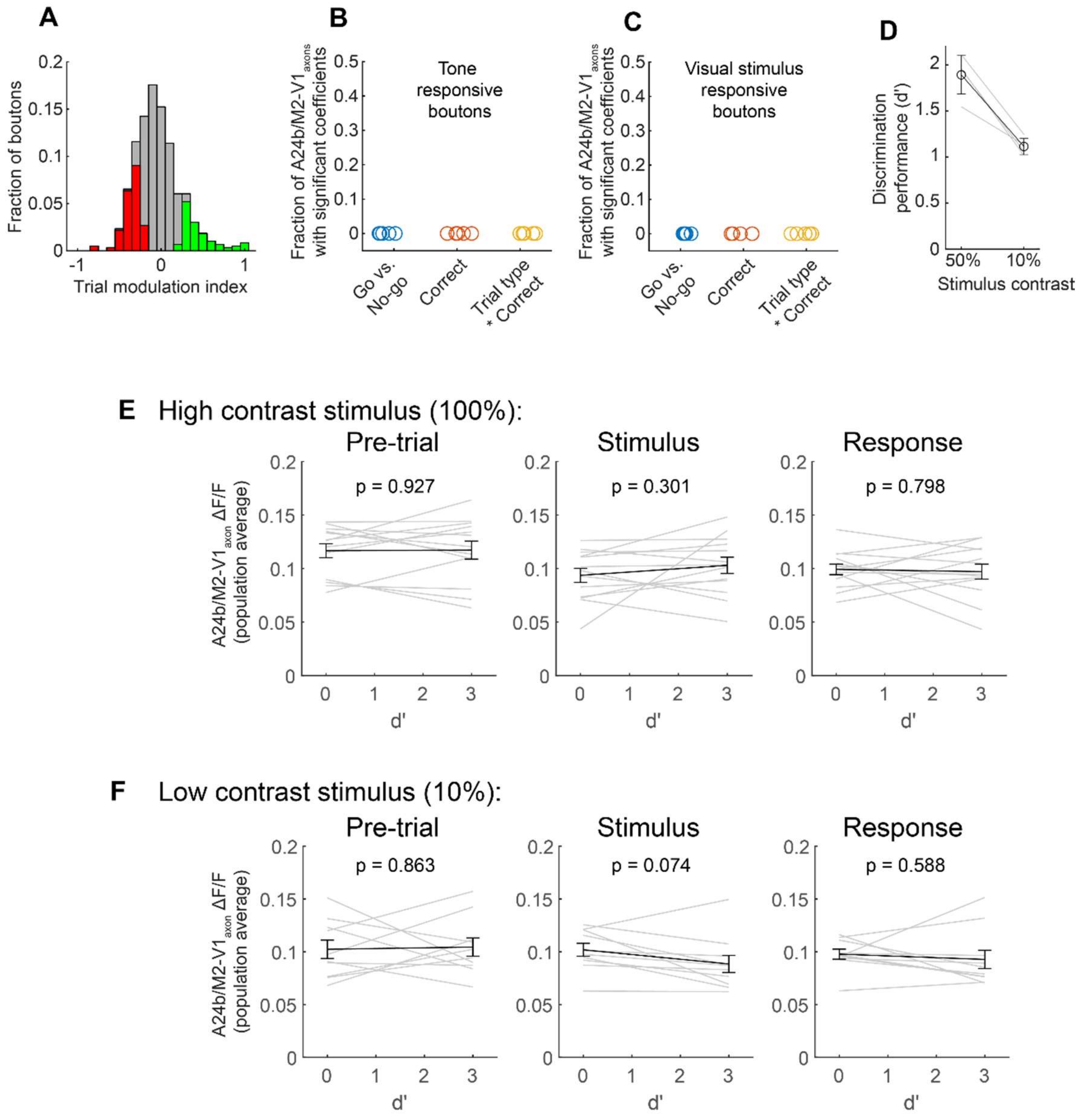

Supplemental Figure 2. Behavioural accuracy is not enhanced by increased A24b/M2-V1$_{axon}$ activity. Related to Figure 1 and 2. (A) The trial modulation index of individual A24b/M2-V1$_{axons}$ showing that there are boutons that are both positively and negatively modulated by whether the animal is within vs. between trials. 597 boutons from 5 behavioural sessions from 5 mice. (B) Behavioural accuracy (correctness) is not associated with level of activity of A24b/M2-V1$_{axons}$ (i.e. ΔF/F) in tone responsive boutons. (C) As in (B) but for visually responsive boutons. (D) Discrimination accuracy reduces at lower compared to higher stimulus contrasts. (E) We observe no relationship between A24b/M2-V1axon ΔF/F and discrimination performance during the task (d’), during either the pre-trial, stimulus period or response period. Grey lines represent linear regression fits to A24b/M2-V1axon ΔF/F vs. d’ in individual experiments while the black lines represent the average of these fits. P values indicate whether the slope of the fits deviate significantly from zero, as would be the case if there was a relationship between these variables. (F) As in (B) but at lower contrast (10%).

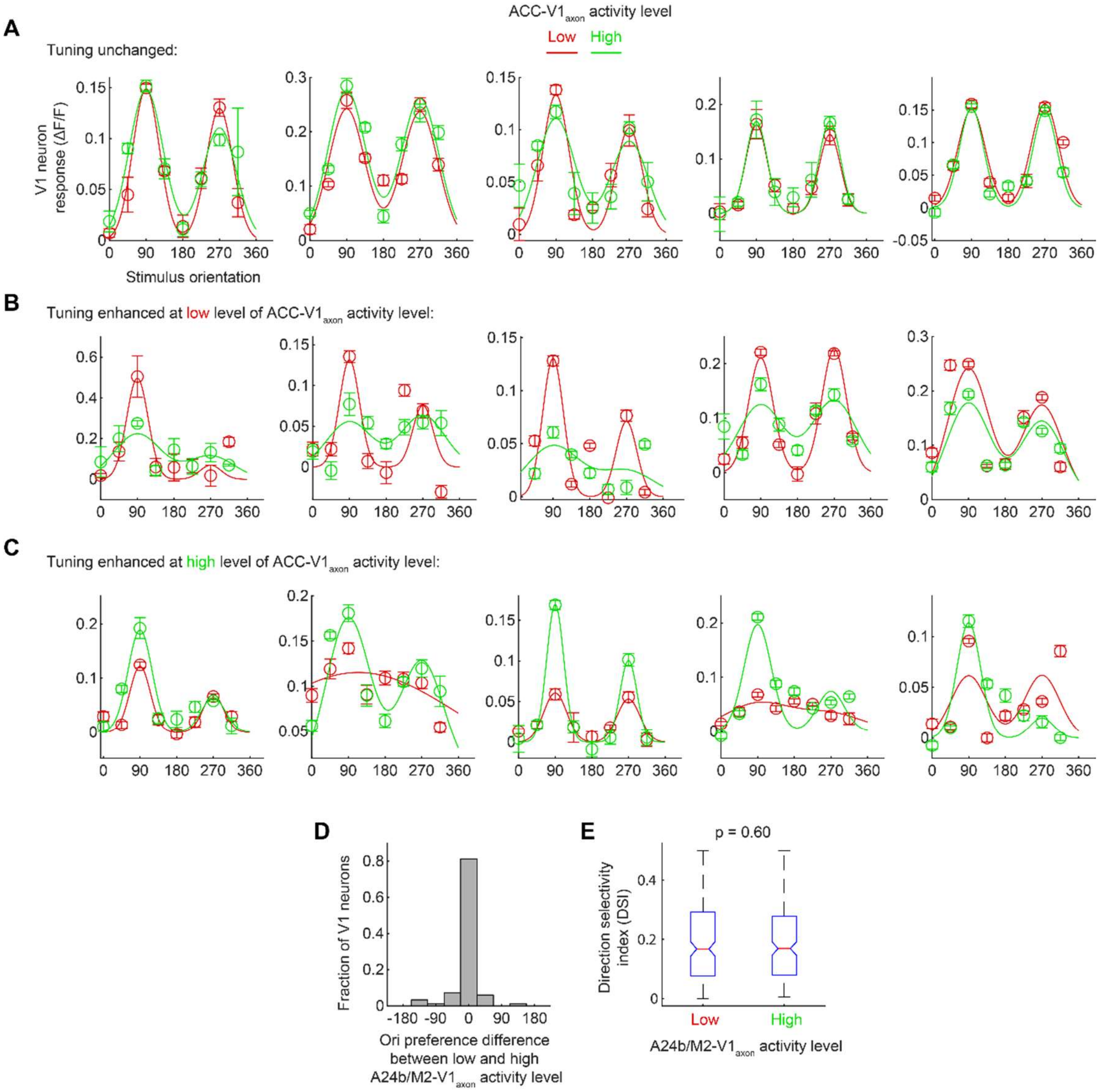

Supplemental Figure 3. Neural sensory discrimination is not enhanced by increased A24b/M2-V1$_{axon}$ activity. Related to Figure 2. (A-C) Examples of orientation tuning curves of individual V1 neurons at high (green) or low (red) levels of activity of A24b/M2-V1$_{axons}$ for neurons in which orientation selectivity was unchanged (A), decreased (B) or increased (C) at higher vs. lower levels of A24b/M2-V1$_{axon}$ activity. (D) Orientation tuning preference did not generally change at higher vs. lower levels of A24b/M2-V1$_{axon}$ activity. (E) Direction selectivity does not on average differ significantly between trials with low compared to high levels of A24b/M2-V1axon activity. Comparison of DSI in (E) was made using a Kruskal–Wallis test (n = 192 cells, from 11 experiments from 3 mice). Analysis of directional tuning was limited to cells which were classified as visually responsive (one-way ANOVA over all stimulus conditions), and for which the R^2 of orientation tuning curve fits (at both low and high levels of A24b/M2-V1axon activity) was > 0.3.

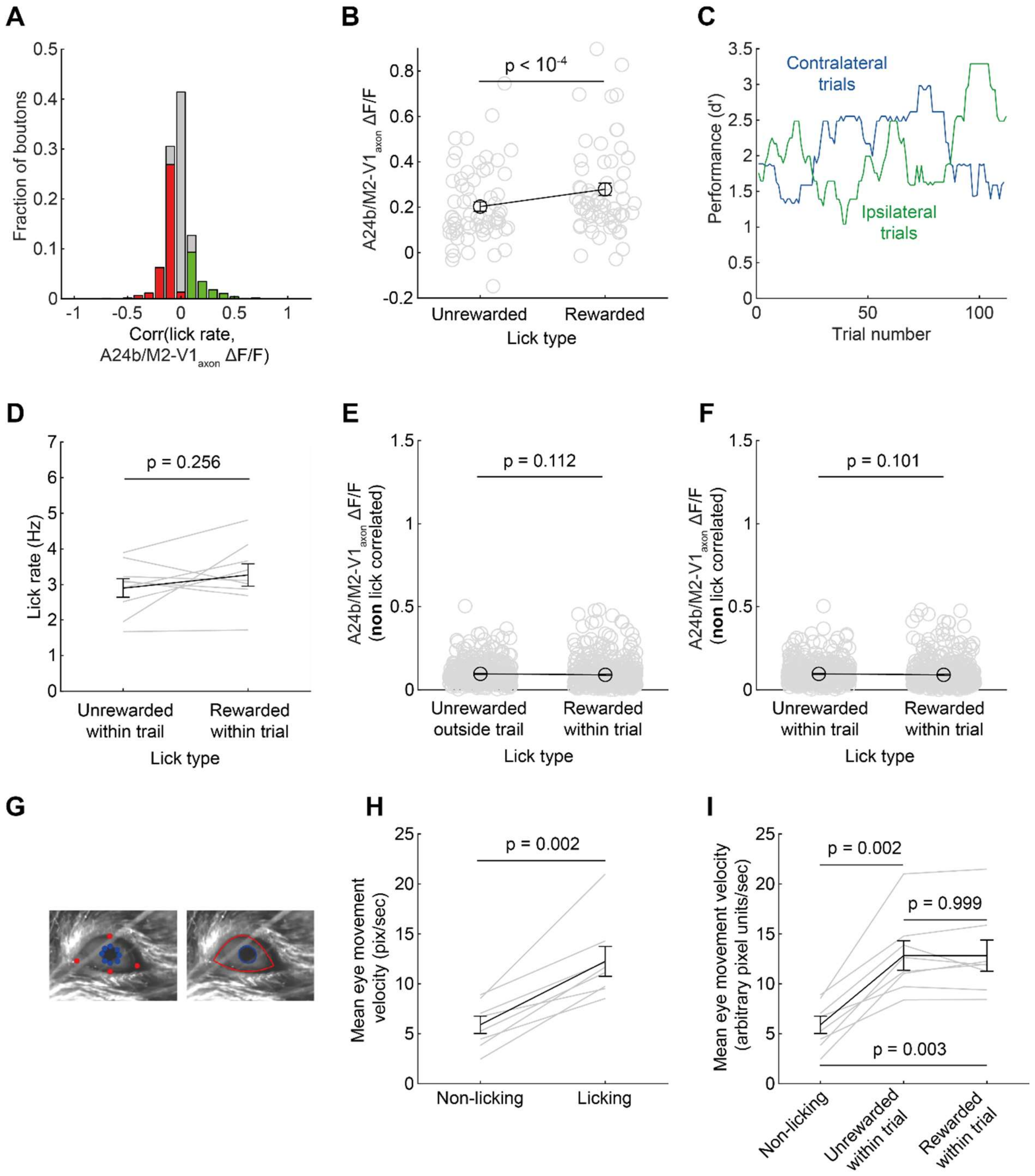

Supplemental Figure 4. A subset of A24b/M2-V1$_{axons}$ show lick correlated activity which is modulated by reward. Related to Figure 3. (A) Distribution of lick correlations of A24b/M2-V1$_{axons}$. 597 A24b/M2-V1axons, from 5 behavioural sessions from 5 mice. (B) Rewarded licks are associated with greater activity in A24b/M2-V1$_{axon}$ activity than unrewarded licks after licking pattern is controlled for. See main text and methods for description of methods used to match patterns of licking. n = 64 boutons from 5 experiments from 5 mice. (C) In some experiments accuracy on ipsilateral vs. contralateral trials varies independently suggesting hemisphere specific variations. (D) The difference between lick rate in the rewarded vs. unrewarded period of the trial was not statistically significantly different (paired sample t-test). (E) In non-lick correlated A24b/M2-V1$_{axons}$ no significant difference was observed between activity during unrewarded licking in the inter-trial period and rewarded licking during go trials. (F) As in (E) except unrewarded licking is analysed during the first 2 seconds of trials during which licking was not rewarded. (G) Left image show one frame of eye video with features detected during DeepLabCut labelled in red (eye corners and upper and lower eyelid) and blue (8 points defining the pupil). Right image shows fits to these

features to define eyelid margins (red; fit with two parabolas) and pupil (blue; ellipse). (H) Mean eye movement velocity was greater during licking as compared to non-licking periods (tested with paired sample two-sided t-test). (I) Mean eye movement velocity was similar between rewarded and unrewarded within trial periods. n = 8 experiments from 7 mice.

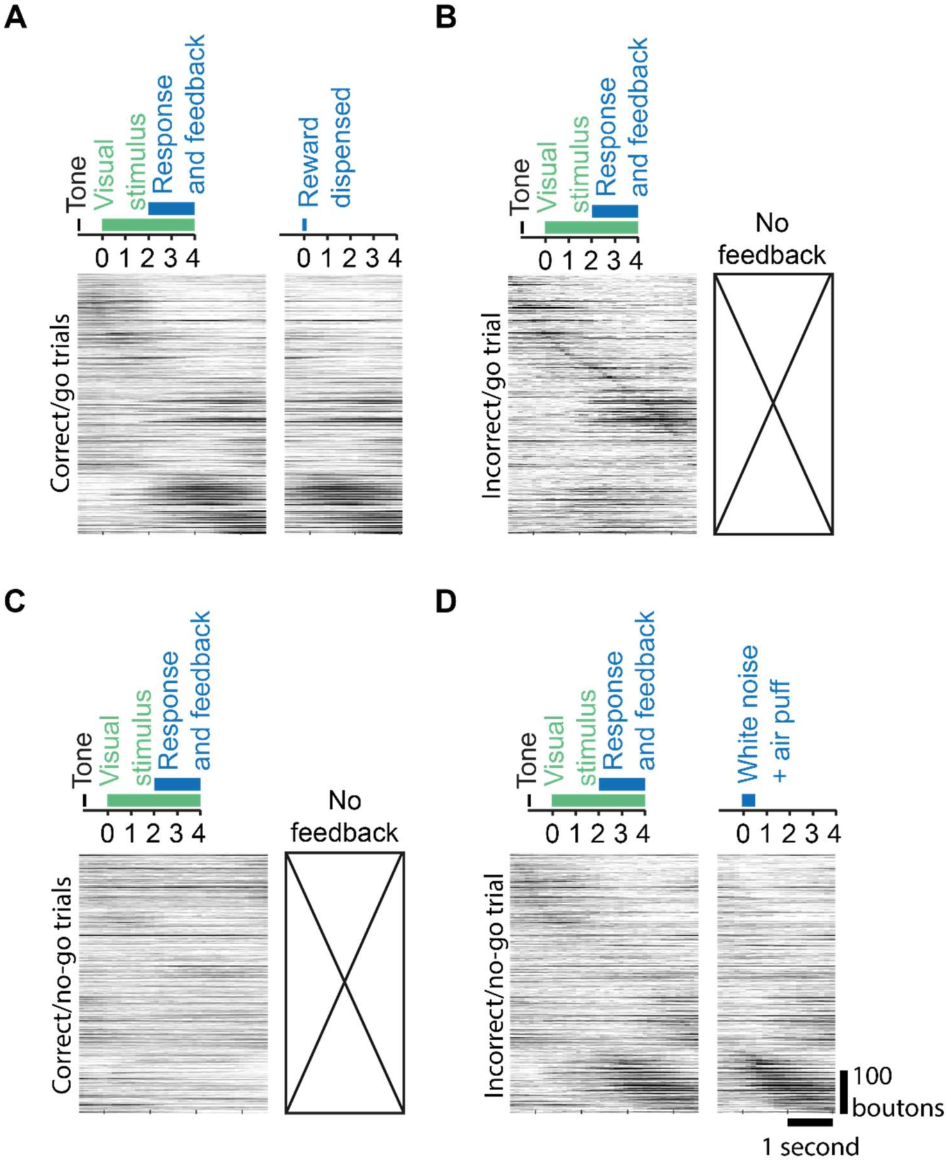

Supplemental Figure 5. Average responses of all A24b/M2-V1$_{axons}$ to all trial events. Related to Figure 1 and 2. (A) Average response of all A24b/M2-V1$_{axons}$ to all trial events in correct/go trials. Each row is a single axon. The left panel show responses to event types common to all trial types, and the right panel shows trial type specific events where appropriate (i.e. feedback). Rows are sorted by the time of maximum mean activity across all 4 trial types. (B-D) As in (A) except for incorrect/go (B), correct/no-go (C) and incorrect /no-go (D) trial types. n = 597 boutons from 5 behavioural sessions from 5 mice.

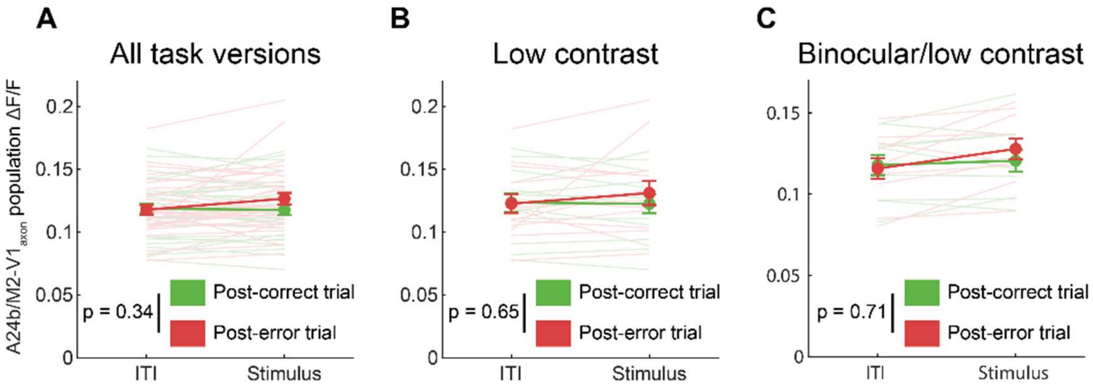

Supplemental Figure 6. Post-error recruitment of A24b/M2-V1$_{axons}$ is not observed under the conditions of our study. Related to Figure 1 and 2. A recent study reported V1 projecting cingulate neurons are selectively activated at a population level following errors during a freely moving 5-choice serial reaction time task (Norman et al., 2021a). We thus tested for activation following errors in our dataset. (A) Comparison of mean ΔF/F of boutons in each experiment post-error or post-correct trial, either during the intertrial period (ITI) or during the stimulus period of the trial. All task versions are included in analysis. Two-way ANOVA test of the effect of previous trial correctness: p = 0.34. n = 32 experiments from 11 mice. (B) As in (A) for low contrast experiments which are of higher difficulty (accuracy is reduced). Two-way ANOVA test of the effect of previous trial correctness: p = 0.65. n = 13 experiments from 7 mice. (C) As in (A) but for bilateral low contrast experiments. In this condition animals do not know in advance where the stimulus will appear, and it is of low contrast, and this is therefore arguably the most difficult version of the task. Two-way ANOVA test of the effect of previous trial correctness: p = 0.71. n = 10 experiments from 5 mice.
