# Supplementary material for: Recruitment of frontal sensory circuits during visual discrimination
Source: Cell Rep. 2022 Jun 7;39(10):110932. doi: 10.1016/j.celrep.2022.110932 (PMC9247711; doi:10.1016/j.celrep.2022.110932)
Supplement: Document S2. Article plus supplemental information [file mmc2.pdf]

Cell Reports

Recruitment of frontal sensory circuits during visual discrimination

Graphical abstract

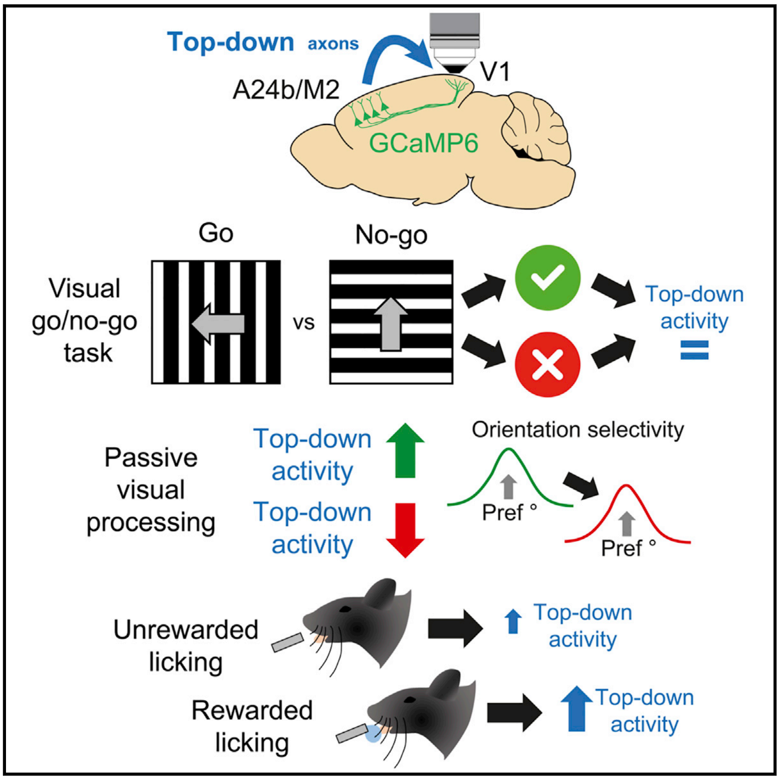

Highlights

- Endogenous A24b/M2-V1 axon activity elevation does not enhance sensory processing
- A24b/M2-V1 axon activity is correlated with licking
- A24b/M2-V1 axon lick-correlated activity is modulated by reward

Authors

Eluned Broom, Vivian Imbriotis, Frank Sengpiel, William M. Connelly, Adam Ranson

Correspondence

aranson@uic.es

In brief

Broom et al. test the link between increased endogenous A24b/M2-V1 axon activity and enhanced visual processing and visually guided behavior. They find no evidence of this association and instead observe that A24b/M2-V1 axon activity is correlated with licking and modulated by reward.

Broom et al., 2022, Cell Reports *39*, 110932
June 7, 2022 © 2022 The Authors.
https://doi.org/10.1016/j.celrep.2022.110932

CellPress

Cell Reports

CellPress
OPEN ACCESS

Report

Recruitment of frontal sensory circuits during visual discrimination

Eluned Broom,[1] Vivian Imbriotis,[2] Frank Sengpiel,[1,3] William M. Connelly,[2,6,7] and Adam Ranson[1,3,4,5,6,7,*]
[1]School of Biosciences, Cardiff University, Cardiff, UK
[2]School of Medicine, University of Tasmania, Hobart, TAS, Australia
[3]Neurosciences & Mental Health Research Institute, Cardiff University, Cardiff, UK
[4]Faculty of Medicine and Health Sciences, Universitat Internacional de Catalunya, Barcelona, Spain
[5]Institut de Neurociències, Universitat Autònoma de Barcelona, Bellaterra, Spain
[6]Senior author
[7]Lead contact
*Correspondence: aranson@uic.es
https://doi.org/10.1016/j.celrep.2022.110932

SUMMARY

A long-range circuit linking the medial frontal cortex to the primary visual cortex (V1) has been proposed to mediate visual selective attention in mice during visually guided behavior. Here, we use *in vivo* two-photon functional imaging to measure the endogenous activity of axons of A24b/M2 neurons from this region projecting to layer 1 of V1 (A24b/M2-V1$_{axons}$) in mice either passively viewing stimuli or performing a go/no-go visually guided task. We observe that while A24b/M2-V1$_{axons}$ are recruited under these conditions, this is not linked to enhancement of neural or behavioral measures of sensory coding. Instead, A24b/M2-V1$_{axon}$ activity is associated with licking behavior, modulated by reward, and biased toward the sensory cortical hemisphere representing the stimulus currently being discriminated.

INTRODUCTION

Sensory processing is powerfully modulated by contextual and behavioral factors such as prior experience, anticipation, attention, and movement (Ayaz et al., 2013; Keller et al., 2012, 2017; Leinweber et al., 2017; Morimoto et al., 2021; Niell and Stryker, 2010; Pakan et al., 2018; Poort et al., 2015; Ranson, 2017; Saleem et al., 2013, 2018; Speed et al., 2020). One mechanism of this modulation is thought to be long-range glutamatergic corticocortical circuits (Gilbert and Li, 2013; Morimoto et al., 2021). In mice, axons originating in higher cortical regions, such as the frontal, retrosplenial, and parietal cortex, and terminating preferentially in layer 1 of early sensory areas such as V1, are thought to influence sensory processing both directly, through excitatory synapses onto the tuft dendrites of V1 pyramidal neurons, and indirectly, through several classes of inhibitory neurons, which in turn modulate excitatory neurons (Makino and Komiyama, 2015; Zhang et al., 2014).

One such circuit in mice monosynaptically connects medial frontal cortical regions (including areas referred to as the anterior cingulate cortex [ACC], A24b and M2) to the primary visual cortex (Zhang et al., 2014) and has been demonstrated to exert a retinotopically selective influence on sensory processing that shares similarities with some forms of selective visual attention described in primates (Armstrong et al., 2006; Moore and Armstrong, 2003; Reynolds and Heeger, 2009; Sundberg et al., 2009). In particular, parallels have been drawn between this medial frontal-originating circuit in mice and gaze-control-associated neurons in the non-human primate frontal eye fields, which also transmit spatially specific selection signals to the sensory cortex (Armstrong et al., 2006; Knudsen, 2007; Moore and Armstrong, 2003; Moore and Zirnsak, 2017). Analogous spatially specific top-down modulatory circuits have been identified in the avian brain, where low-level stimulation of the arcopallial gaze field (the avian frontal eye field equivalent) results in spatially specific alterations of the responsiveness of auditory optic tectum neurons (Knudsen, 2007; Winkowski and Knudsen, 2006).

We refer here to axons constituting the medial frontal to V1 projection in mouse as A24b/M2-V1$_{axons}$. Previous experiments have found that optogenetic activation of A24b/M2-V1$_{axons}$ enhances the specificity of V1 neuron orientation tuning and improves behaviorally measured stimulus orientation discrimination (Zhang et al., 2014). A limitation of this previous work is that, although it has demonstrated that this circuit could in principle function to enhance sensory processing, the relevance of the artificially induced patterns of circuit activation to normal physiological function remains uncertain. Specifically, direct evidence of endogenously generated increased activity of A24b/M2-V1$_{axons}$ being linked to improved behavioral or neuronal stimulus discrimination is lacking. In addition, subsequent studies have argued for somewhat different functions for axons originating in overlapping regions of the cingulate cortex, including in elevation of attention following errors during a freely moving five-choice serial reaction time task (Norman et al., 2021a; 2021b), in sensory motor integration (Huda et al., 2020), in relaying locomotion-driven motor signals to V1 (Leinweber et al., 2017), and in mediating spatial visual expectation (Fiser et al., 2016). A further caveat associated with linking the

This is an open access article under the CC BY license (http://creativecommons.org/licenses/by/4.0/).

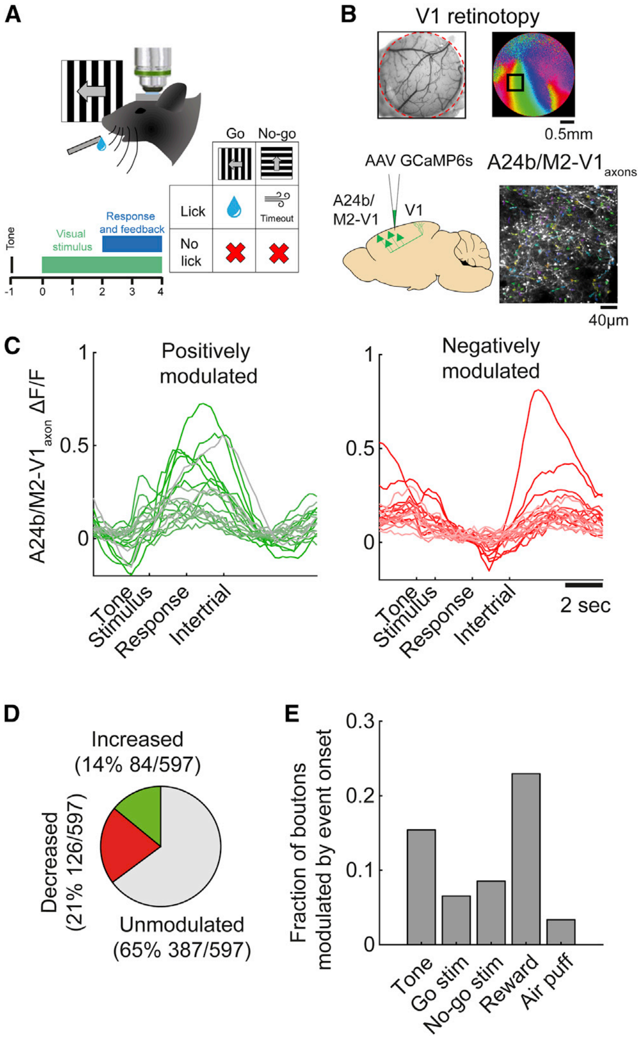

Figure 1. A24b/M2-V1$_{axons}$ are recruited during go/no-go visual discrimination behavior

(A) Schematic of monocular go/no-go visual discrimination task.

(B) Visualization of the location of the monocular primary visual cortex using intrinsic signal imaging and labeling of A24b/M2-V1$_{axons}$ by injection into A24b/M2 followed by visualization using *in vivo* two-photon microscopy in monocular V1 after 2–4 weeks.

(C) Example boutons that were positively (green) or negatively (red) modulated by whether the animal was within a trial or in the intertrial period. Each line represents an individual bouton and is an average of 253 trials.

(D) Fraction of boutons that exhibited increased, decreased, or unchanged activity when a comparison was made between within-trial period activity and intertrial period activity. We assessed 597 boutons from five behavioral sessions from five mice; paired-sample t test, $p < 0.01$.

(E) Fractions of the same boutons that responded with significantly increased activity to different types of trial events.

proposed attentional function of this medial frontal circuit in rodents to visual attentional mechanisms in primates is the broader question of the homology between structures in the rodent and primate frontal cortices (Laubach et al., 2018; Passingham and Wise, 2012). In primates, for example, anterior regions of the cingulate cortex have been more closely linked to aspects of executive behavioral control, such as error detection (Carter et al., 1998), reward-based decision-making (Bush et al., 2002), and control of action selection (Shenhav et al., 2013) than to selective visual attention. Conversely, stimulation of medial frontal cortical regions in rodents elicits head-orienting behavior, supporting the notion of a functional similarity to the frontal eye fields (Sinnamon and Galer, 1984).

Here, we aimed to clarify the proposed attentional function of the A24b/M2-V1 circuit in mice by reproducing the go/no-go head-fixed behavioral paradigm employed by Zhang et al. (2014), but measuring at single-axonal-bouton resolution the endogenous recruitment of A24b/M2-V1$_{axons}$ and their relationship to behavioral and neural stimulus discrimination. In this context we find no evidence of an association between endogenous recruitment of this circuit and enhanced behavioral or neural measures of stimulus discrimination. Instead, we observe strong recruitment of the circuit in a subset of boutons by licking motor behavior that is modulated by whether licking is rewarded and biased toward the hemisphere currently processing task-relevant sensory signals.

RESULTS

We first sought to assess previous claims of a role for long-range corticocortical projections from the cingulate cortex to the primary visual cortex (V1) in exerting top-down modulation of V1 activity that can enhance behavioral and neural visual discrimination accuracy (Zhang et al., 2014). While optogenetic activation of A24b/M2-V1$_{axons}$ during a go/no-go orientation discrimination task was previously shown to enhance discrimination accuracy, we aimed to evaluate if this circuit is also endogenously recruited in this way during visual processing.

A24b/M2-V1$_{axons}$ are endogenously recruited during visual discrimination

A24b/M2-V1$_{axons}$ were labeled using the genetically encoded calcium indicator GCaMP6s (Chen et al., 2013) and imaged using two-photon microscopy in layer 1 of V1 (Figures 1A, 1B, and S1A) in animals trained to perform a go/no-go stimulus orientation discrimination task at a high level of accuracy (discrimination as quantified by $d' > 1.5$). We first tested whether the activity ($\Delta F/F$) of A24b/M2-V1$_{axons}$ differed between within-trial periods (when the animal was actively engaged in discrimination) and intertrial periods. We found that activity was significantly higher during discrimination versus intertrial periods in 14% of A24b/M2-V1$_{axons}$ (Figures 1C and 1D; 84/597 from five behavioral sessions from five mice; paired-sample t test), suggesting that the go/no-go behavior results in recruitment of this circuit. A further 21% of A24b/M2-V1$_{axons}$ (Figures 1C and 1D; 126/597) exhibited the opposite behavior of higher levels of activity during intertrial periods, suggesting suppression of activity during task engagement reminiscent of the findings of a recent study from the ACC population more broadly (Kim et al., 2021). The distribution of the degree of trial modulation of all boutons is shown in Figure S2A, quantified with an index of trial modulation. We next tested for transient increases of A24b/M2-V1$_{axon}$ activity following specific stimulus events within the task. This showed that subsets of

$A24b/M2\text{-}V1_{axons}$ exhibit elevated activity in response to pre-trial tone, go and no-go stimulus onset, reward administration, and air puff (15.4%, 6.5%, 8.5%, 22.9%, and 3.4% of boutons, respectively; Figure 1E; see also Figure S5 for averaged responses of all boutons during different task phases). Thus, the activity of some $A24b/M2\text{-}V1_{axons}$ was observed to vary systematically during the visual discrimination task, and these modulations of activity happened during various task phases.

Endogenous $A24b/M2\text{-}V1_{axon}$ activity does not co-vary with behaviorally reported visual discrimination

Optogenetic activation of $A24b/M2\text{-}V1_{axons}$ has been shown to enhance visual discrimination (Huda et al., 2020; Zhang et al., 2014), and so we next asked whether enhanced discrimination accuracy was also associated with endogenously elevated activity of $A24b/M2\text{-}V1_{axons}$. Within each behavioral session there was slow variation in accuracy over timescales of minutes (i.e., fluctuations in hit rate and false alarm rate and consequently d′; Figures 2A and 2B), suggesting varying levels of arousal, attention, or task engagement. To test the association between $A24b/M2\text{-}V1_{axon}$ activation and this fluctuating discrimination accuracy, we fit a linear model to predict average bouton activity in each trial, using trial correctness and trial type (go or no-go) as predictors. A statistically significant association between $A24b/M2\text{-}V1_{axons}$ activity and trial correctness was observed in only a small fraction of boutons (5.7%; 34/597 $A24b/M2\text{-}V1_{axons}$, from five behavioral sessions from five mice; Figure 2C), and of these, 47% were more active during correct trials and 53% were less active during correct trials (Figure 2C, inset). We next tested if there might be subpopulations of boutons that respond to stimulus events in the task and show variations in these responses depending upon task correctness. We used the same model to test if tone- and visual stimulus-responsive boutons (shown in Figure 1E) showed differences in their activity after these stimulus events depending on trial correctness but found no such differences (Figures S2B and S2C). These results suggest that endogenous activation of $A24b/M2\text{-}V1_{axons}$ is unlikely to be playing a significant role in enhancing visual discrimination in this behavioral context.

In the trials analyzed above, the stimulus contrast was maximum and discrimination accuracy was high (mean $d' = 1.67$). We hypothesized that this could cause a ceiling effect whereby improvements in stimulus discrimination due to top-down modulations in the efficacy of V1 encoding are limited, or that the circuit may be recruited only when task demands are high (Bahrami et al., 2007; Norman et al., 2021b). We tested this possibility in a different group of animals in which task difficulty was increased by altering stimulus contrast in a subset of trials. As expected, discrimination accuracy decreased at lower contrasts (Figure S2D; high contrast [50%] $d' = 1.89 \pm 0.12$; low contrast [10%] $d' = 1.11 \pm 0.05$; $n = 4$ mice; $p = 0.001$; paired-sample t test). We again tested whether cingulate-axon activity was elevated on correct versus incorrect trials when the task was more difficult. This analysis also showed that almost no cingulate-axons (<0.1%) showed activity that differed significantly between correct and incorrect trials (Figure 2D). Finally, we assessed whether behavioral errors might instead drive recruitment of $A24b/M2\text{-}V1_{axons}$, as has previously been shown (Norman et al., 2021a); however, $A24b/M2\text{-}V1_{axon}$ activity was also not found to differ significantly as a function of previous trial correctness (see Figure S6 and supplemental information). Together, these results indicate the lack of association between endogenous cingulate axon activity, and discrimination accuracy is unlikely to be due to task difficulty in the context of this task.

Endogenous $A24b/M2\text{-}V1_{axon}$ activity does not co-vary with neural stimulus discrimination

As well as having effects on behaviorally reported stimulus orientation discrimination, optogenetic activation of $A24b/M2\text{-}V1_{axons}$ has also been shown to enhance orientation tuning in V1 during passive viewing by enhancing responses at the preferred orientation (Zhang et al., 2014). These findings prompted us to ask if V1 encoding might be enhanced by increased endogenous activity of $A24b/M2\text{-}V1_{axons}$, even in the absence of behavior enhancement. In support of this possibility, a disassociation has been reported between visual stimulus encoding fidelity in V1 in mice and behavioral readouts of visual discrimination, whereby neural encoding precision of sensory stimuli significantly exceeds that measured behaviorally (Stringer et al., 2021). We used multiplane imaging and red and green calcium indicators to concurrently measure the activity of A24b/M2-$V1_{axons}$ in layer 1 (labeled green with GCaMP6s-axon; Broussard et al., 2018) and V1 neuron somas (labeled red with jRGECO1a; Dana et al., 2016) in layers 2/3 during passive visual stimulation with drifting gratings (Figures 2E and 2F). We first tested whether orientation selectivity improved with increased endogenous population $A24b/M2\text{-}V1_{axon}$ activity as might be anticipated from previous findings of artificially activating this feedback pathway. Mean population activity of $A24b/M2\text{-}V1_{axons}$ varied significantly during the course of each experiment ($64 \pm 0.04\%$ difference between the 20th and the 80th percentile), allowing us to assess stimulus orientation selectivity of V1 at different levels of $A24b/M2\text{-}V1_{axon}$ population activity. An orientation selectivity index (OSI) was calculated for each V1 neuron using data from trials when $A24b/M2\text{-}V1_{axon}$ activity was either high or low (upper or lower 50% of $A24b/M2\text{-}V1_{axon}$ activity levels). Inconsistent with an association between endogenous A24b/M2-$V1_{axon}$ activity and orientation tuning, orientation selectivity was not enhanced on trials with higher compared with lower levels of $A24b/M2\text{-}V1_{axon}$ activity (Kruskal-Wallis test; $n = 192$ V1 neurons, from 11 experiments from three mice; $p = 0.91$; Figures 2G and 2H). The same analysis, but of direction selectivity (quantified using an index of direction selectivity, DSI), also showed no significant enhancement with increased endogenous $A24b/M2\text{-}V1_{axon}$ activity (Kruskal-Wallis test; $p = 0.60$; Figure S3E). Individual cells were found to exhibit shifts in orientation tuning selectivity as a function of increased A24b/M2-$V1_{axon}$ activity, but across the population these shifts were not systematically biased toward sharpening or broadening of tuning (see Figures S3A–S3C for examples), and overall orientation preference was unchanged between high and low levels of $A24b/M2\text{-}V1_{axon}$ activity (Figure S3D). Overall, these findings are inconsistent with the idea that endogenous fluctuations in $A24b/M2\text{-}V1_{axon}$ activity enhance orientation or direction selectivity in V1.

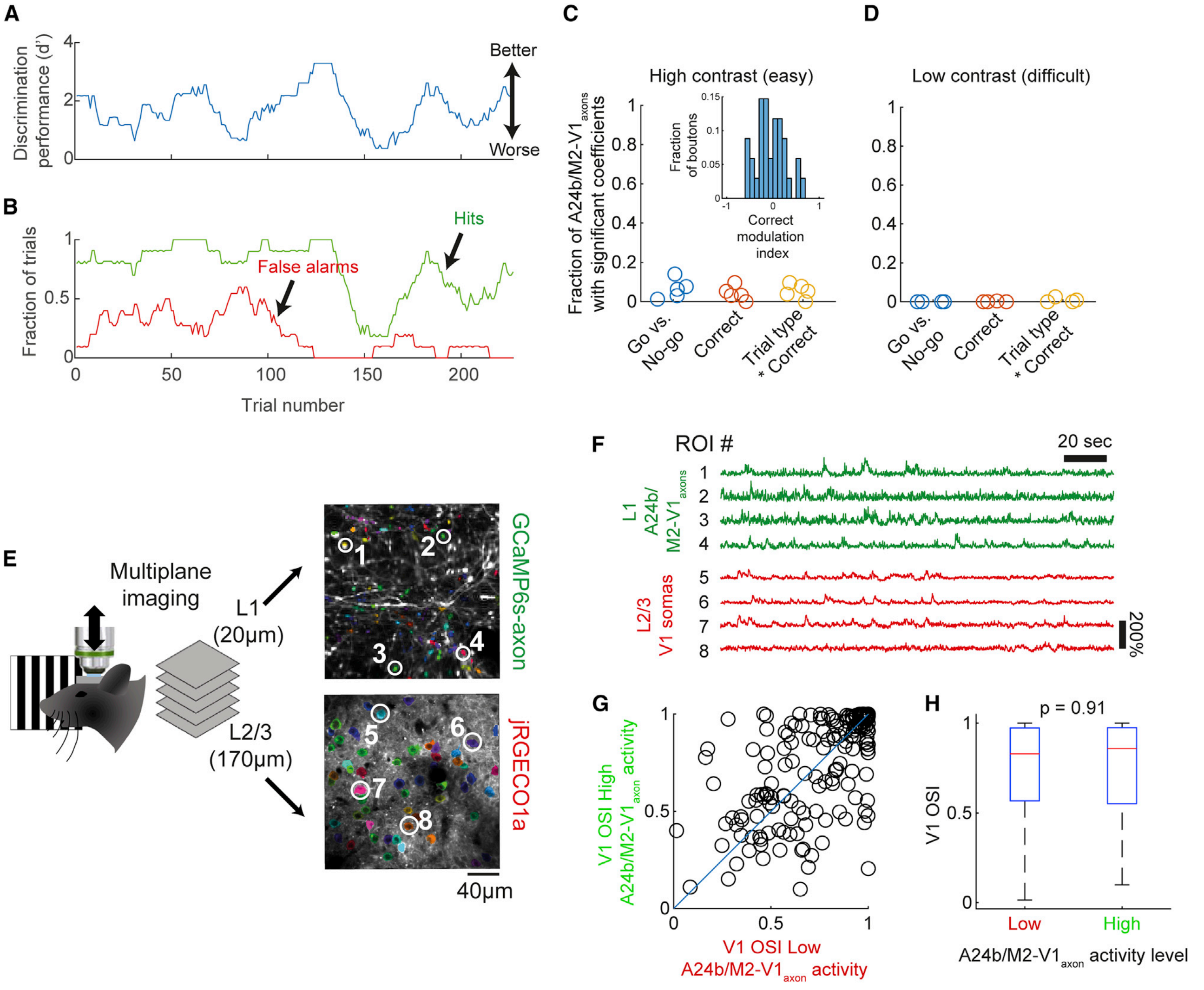

Figure 2. Behavioral accuracy and neural sensory discrimination are not enhanced by increased A24b/M2-V1$_{axon}$ activity

(A) Discrimination accuracy fluctuates markedly within each behavioral session as quantified by d′.

(B) Discrimination accuracy fluctuation is driven by variation in both hit and false alarm rates.

(C) Behavioral accuracy (correctness) is not associated with level of activity of A24b/M2-V1$_{axons}$ (i.e., ΔF/F) under high contrast (i.e., easier) stimulus conditions. We assessed 597 A24b/M2-V1 boutons from five behavioral sessions from five mice.

(D) As in (C), but at low stimulus contrast and with comparable findings of a lack of evidence of association between accuracy and level of activity of A24b/M2-V1$_{axons}$.

(E) Schematic of multiplane imaging to concurrently record cingulate axons in layer 1 (labeled green with axon-GCaMP6s) and V1 neurons in layers 2/3 (labeled red with jRGECO1a).

(F) Example traces of concurrently recorded boutons (green, numbered 1 to 4) and somas (red, numbered 5 to 8).

(G and H) Orientation selectivity does not on average differ significantly between trials with low compared with high levels of A24b/M2-V1$_{axon}$ activity. Comparison of OSI in (H) was made using a Kruskal-Wallis test (n = 192 V1 neurons, from 11 experiments from three mice). Analysis of orientation tuning curves was limited to cells that were classified as visually responsive (one-way ANOVA over all stimulus conditions) and for which the R^2 of the orientation tuning curve fits (at both low and high levels of A24b/M2-V1$_{axon}$ activity) was >0.3. Box plots in (H) show first and third quartile (box), maximum and minimum values (whiskers), and median (red horizontal line).

A24b/M2-V1$_{axon}$ activity is associated with rewarded licking

A subset (17%) of A24b/M2-V1$_{axons}$ showed statistically significant licking-correlated activity, which explained some of the recruitment of these axons during the discrimination phase of the task (tested with permutation test with 1,000 shuffled lick rate traces; $p < 0.05$; Figures 3A and S4A). As licking often co-occurred with reward, we sought to disambiguate these two factors. Licking was often not rewarded (i.e., during intertrial periods, in no-go trials, and during the initial 2 s of stimulus presentation of the trial), allowing a comparison of A24b/M2-V1$_{axon}$ recruitment between rewarded

CellPress
OPEN ACCESS

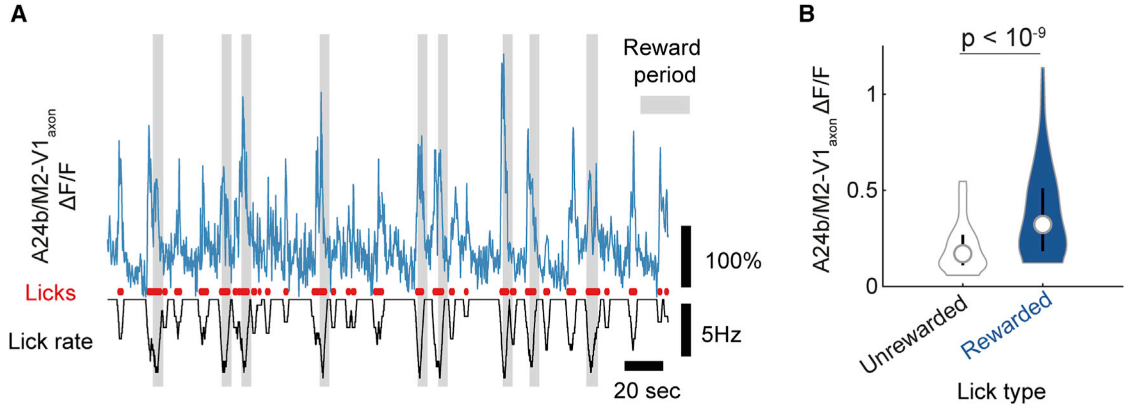

Figure 3. Licking-associated activity of A24b/M2-V1$_{axons}$ is modulated by reward

(A) Example traces of lick-correlated bouton (blue), individual lick events (red), and lick rate (black; more negative indicates higher lick rate). Gray areas show periods when licking is rewarded.

(B) In lick-correlated boutons, rewarded licking (during the reward period of go trials) results in a higher level of activity (ΔF/F) than unrewarded licking (during the initial 2 s of visual stimulus during go trials). Paired-sample t test; $p < 10^{-9}$; n = 64 boutons from five experiments from five mice. Vertical lines in violin plot indicate first and third quartile, and circle symbol indicates median.

and non-rewarded licking. We compared the ΔF/F of highly lick-correlated boutons (R > 0.2) during rewarded licking (within the go trial reward period) with unrewarded licking (during the initial 2 s of stimulus presentation of the go trials) and found that almost all boutons were more active during rewarded versus unrewarded licking, suggesting that licking-associated A24b/M2-V1$_{axon}$ activity encodes a combination of both licking and reward (unrewarded versus rewarded ΔF/F = 0.21 ± 0.02 versus 0.38 ± 0.03; $p < 10^{-9}$; paired-sample t test; n = 64 boutons; Figure 3B). As expected, this relationship was not observed in non-lick-correlated boutons (Figures S4E and S4F) and persisted after lick-associated eye movements and lick frequency were controlled for (Figures S4D and S4G–S4I).

Rewarded licking patterns tended to be longer lasting and more rhythmic (typically > 6 Hz) than non-rewarded licking, which could result in a difference in neural activity unrelated to reward *per se*. To control for this, we identified periods of rewarded and unrewarded rhythmic licking at > 6 Hz. Unrewarded licking bouts were typically shorter than bouts of rewarded licking, and so we limited analysis to A24b/M2-V1$_{axon}$ activity during the first three licks in a bout of three or more licks. For this analysis a bout of licking was thus defined as three licks that occurred within 0.5 s that was preceded by at least 1 s during which no licking occurred; an average of 6% of rewarded licks and 13% of unrewarded licks fall into this constrained definition. Consistent with the previous analysis, we found that frequency- and count-matched licking bouts were associated with greater A24b/M2-V1$_{axon}$ activity when rewarded than when unrewarded (unrewarded versus rewarded ΔF/F = 0.19 ± 0.01 versus 0.37 ± 0.029; $p < 10^{-8}$; paired-sample t test; Figure S4B). This result indicates that differences in rewarded versus unrewarded licking are unlikely to be explained by licking pattern. These data suggest that A24b/M2-V1$_{axon}$ activity encodes in part the presence of reward.

A24b/M2-V1$_{axon}$ licking/reward signals are biased toward task-relevant sensory cortex

We next asked if the licking/reward signals observed are targeted to the areas of the visual cortex processing the sensory signals that are guiding discrimination behavior or, alternatively, are also relayed non-specifically to areas not involved in the discrimination. We trained animals on a variant of the go/no-go task (Figure 4A) in which the stimulus was presented randomly to either the contralateral or the ipsilateral monocular visual field such that on each trial the stimulus was being primarily processed in either V1 in the imaged hemisphere (hemisphere contralateral to the stimulus) or V1 in the non-imaged hemisphere (hemisphere ipsilateral to the stimulus). Animals learned the bilateral version of the task to a high level of accuracy and tended to have similar levels of accuracy when processing visual stimuli via the left and right hemispheres (Figure 4). Fluctuations in task accuracy were most often correlated between the left and the right monocular visual field discrimination trials, suggesting a global cause of fluctuation of accuracy (Figure 4C); however, sometimes fluctuations were side dependent, suggesting possible hemisphere-specific effects (Figure S4C). As observed in the monocular version of the task, a subset of A24b/M2-V1$_{axons}$ showed activity correlated with licking (Figure 4D), and of these, most showed greater activity during rewarded versus non-rewarded licking on both contralateral and ipsilateral stimulus trials (Figure 4E). To test the specificity of the hemispheric targeting of these licking/reward signals, we examined if, on a trial-by-trial basis, rewarded licking signals were preferentially targeted to V1 in the hemisphere where task-related sensory signals were primarily arriving (i.e., the hemisphere contralateral to the monocular stimulus) or alternatively to V1 in both hemispheres. We thus measured mean rewarded ΔF/F in trials that were contralateral or ipsilateral to visual cue presentation and found that they were significantly larger when visual cues were presented contralaterally. To further quantify this bias, we calculated a "reward targeting index" (RTI) whereby values of

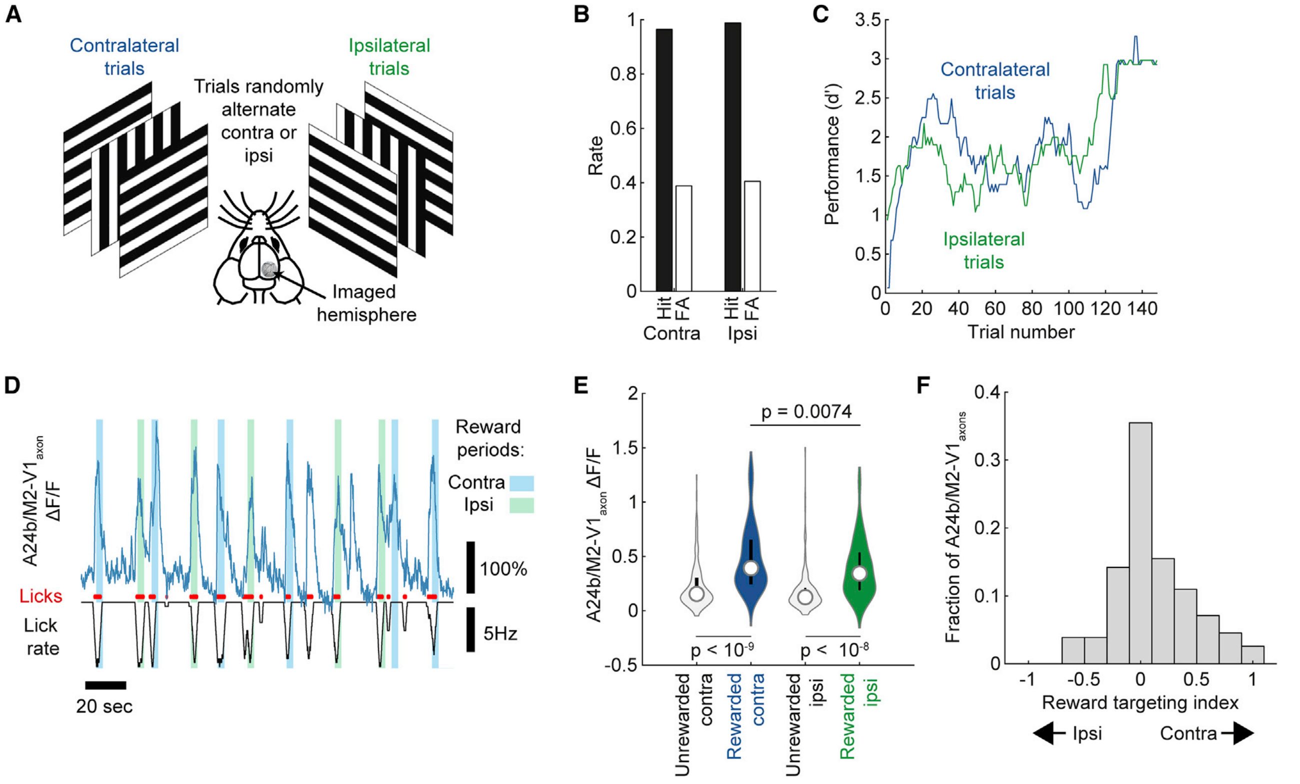

Figure 4. Licking-associated activity in A24b/M2-V1$_{axons}$ is modulated by reward and hemispherically biased depending upon current task conditions

(A) Schematic of bilateral version of the visual discrimination task.

(B) Accuracy was generally similar on ipsilateral and contralateral trials.

(C) During the course of one experiment, contralateral and ipsilateral accuracy often vary together, suggesting a global change in attentional or motivational state.

(D) Example traces of a lick-correlated bouton in the bilateral task, individual lick events (red), and lick rate (black; note more negative indicates higher lick rate). Light blue and light green shaded areas show contralateral and ipsilateral trial reward periods, respectively.

(E) In lick-correlated boutons, rewarded licking results in a higher level of activity (ΔF/F) than unrewarded licking (for contralateral trials, unrewarded ΔF/F = 0.23 ± 0.02, rewarded ΔF/F = 0.48 ± 0.03, $p < 10^{-9}$; for ipsilateral trials, unrewarded ΔF/F = 0.18 ± 0.02, rewarded ΔF/F = 0.39 ± 0.02, $p < 10^{-8}$; n = 155 boutons from 10 experiments from five mice; two-tailed t test after correction for multiple comparisons using the Tukey-Kramer method). This effect is greatest on contralateral trials when the stimulus is being represented in monocular V1 in the same hemisphere as the A24b/M2-V1$_{axons}$ are originating from (p = 0.0074; two-tailed t test after correction for multiple comparisons using the Tukey-Kramer method). Vertical lines in violin plot indicate first and third quartile, and circle symbol indicates median.

(F) Distribution of reward targeting index values is significantly greater than 0, indicating a slight bias in the targeting of lick/reward signals toward the hemisphere contralateral to the side of the visual cue stimulation (60% of boutons had an RTI > 0; two-tailed one-sample t test for difference from 0; p = 0.002).

−1 and 1 respectively indicate that reward signals are exclusively targeted to V1 ipsilateral or contralateral to the stimulus, while a value of 0 indicates equal targeting to the two hemispheres. The median RTI was 0.09, indicating a modest bias in reward signal targeting to V1 in the hemisphere processing the task-related stimulus (Figure 4F). Together, these results indicate that, while licking- and reward-associated signals from A24b/M2-V1$_{axons}$ are relayed to both cue-encoding and non-cue-encoding regions of V1 during this task, they are biased (although weakly) toward areas of V1 encoding the visual cue.

DISCUSSION

Our experiments reveal that, while A24b/M2-V1$_{axons}$ projecting to V1 are endogenously recruited during both passive visual stimulation and the go/no-go visual discrimination task studied, this recruitment is not associated with enhanced neural or behavioral discrimination of stimulus orientation. Instead, we find that a significant fraction of A24b/M2-V1$_{axons}$ exhibit activity that is correlated with licking. In these boutons, licking activity was modulated by whether it was rewarded and, in addition, moderately biased toward the hemisphere receiving task-associated sensory stimuli.

Our findings are at odds with some previous studies in which exogenous optogenetic excitation (Zhang et al., 2014) or suppression (Huda et al., 2020) of the same A24b/M2-V1 projection has been found to respectively enhance orientation discrimination behavior or impair visual detection-driven orienting behavior. Similarly, optogenetic excitation of this circuit has previously been reported to enhance orientation tuning in V1 during passive viewing (Zhang et al., 2014), while in our experiments, endogenously heightened activity in the A24b/M2-V1$_{axon}$

population was not associated with a sharpening of V1 orientation tuning. In this respect, our findings are more consistent with aspects of another report in which fiber photometry was used to measure the summed neural activity of cingulate neurons projecting to V1 in freely moving mice performing a five-choice serial reaction time task (Norman et al., 2021a). This study also found that increased cingulate activity (following errors) was not associated with improved behavioral accuracy on immediately subsequent trials. Together, our findings suggest the possibility that the powerful and synchronous activation/inactivation of this circuit obtained using optogenetic approaches, and the consequent effects on behavior and sensory coding, may be quite different from the range of activity this circuit exhibits endogenously. Our results therefore raise the possibility that, while the A24b/M2-V1 projection in mice may share some organizational similarities with circuits implicated in selective attention in primates (such as exhibiting retinotopic spatial specificity of modulation), its endogenous function may be quite different. Indeed, the signals related to action selection and reward that we have observed suggest a function closer to the executive control of goal-directed behavior than to attention. This possibility is consistent with a recent study in mice that examined the feedforward transmission of information from sensory cortex to anterior cingulate cortex and described a circuit through which visual cortical input to the cingulate cortex gates the initiation of reward-directed licking through feedforward inhibition (Kim et al., 2021). Notably, Kim et al. observed a large fraction of neurons within the ACC population in general that were suppressed during trial engagement compared with intertrial periods, which is consistent with our observations in A24b/M2-V1$_{axons}$. Given this apparent role of ACC in regulating sensory-driven response inhibition, the specific function that might be conferred by retinotopically specific feedback to visual cortex by A24b/M2-V1$_{axons}$ remains unclear.

We examined our dataset for evidence of previously reported post-error recruitment of V1 projecting cingulate neurons (Norman et al., 2021a), but found that even in the more demanding versions of our task there was no evidence for such a pattern of activity (post-error recruitment of the circuit has been reported to be limited to conditions of higher task difficulty; Norman et al., 2021b). One possibility is that our task is not sufficiently cognitively demanding, although we observed a clear reduction in accuracy when reducing stimulus contrast, suggesting we are not at a ceiling accuracy level. There are also likely to be important differences due to details of the specific task, such as whether the animal is freely moving. It would be of interest to develop head-fixed task variants that reproduce the post-error circuit recruitment previously described (Norman et al., 2021a), but with greater control of visual stimulus and motor behavior than that possible in freely moving animals. Finally, there may be subregions of cingulate cortex, not captured in our study, that perform this function.

Other studies have described motor-related activity in A24b/M2-V1$_{axons}$ (Huda et al., 2020; Leinweber et al., 2017), although not, to the best of our knowledge, licking/reward-driven activity (although see Kim et al., 2021, for evidence of such activity in the ACC in general). Leinweber et al. (2017) studied V1 projecting axons from region A24b and argued for a distinct role of the projection in relaying predictions to V1 of visual flow based on motor output. While we have observed clear motor-related activity driven by licking, it is unclear what visually might be predicted to follow as a consequence of licking behavior and how this relates to the apparent reward contingency of this activity. One possible movement-associated visual event in the context of our experiments is that the offset of the stimulus at the end of the trial typically coincides with licking. Longitudinal analysis of A24b/M2-V1$_{axons}$ during task acquisition will help determine if this might be linked to the recruitment of A24b/M2-V1$_{axons}$ observed during licking, as such an association would need to be learned. Another possibility is that the eye movements that reliably occur during licking (Figure S4D) could drive the recruitment of A24b/M2-V1$_{axons}$ relaying visual predictions to V1.

A number of previous studies have identified reward-related activity in V1 neurons (Pakan et al., 2018; Poort et al., 2015; Schuler and Bear, 2009); however, the route through which these signals impinge upon V1 has remained unclear. Here we report A24b/M2-V1$_{axons}$ as a circuit through which reward and licking signals enter V1 in a way that is biased, at least at a gross scale, toward currently engaged regions of sensory cortex. The role that such reward and licking signals play in visually guided behavior remains unclear, but we speculate that they may be involved in driving and maintaining previously described plasticity processes that result in alterations of the representations of previously rewarded sensory stimuli (Poort et al., 2015). Further experiments in which such reward-signal-carrying A24b/M2-V1$_{axons}$ are selectively inactivated will be required to test this possibility.

Limitations of the study

Here we attempted to closely mimic the behavioral conditions of a previous study, which concluded that A24b/M2-V1$_{axons}$ (referred to as cingulate axons in the study in question) exert an influence on V1 akin to visual selective attention described in primates (Zhang et al., 2014). A first limitation of our study is that, although we found no association between endogenous recruitment of A24b/M2-V1$_{axons}$ and behavioral measures of stimulus orientation discrimination, in other behavioral tasks this may not be the case. Although we consider that we could reasonably expect to expose activity of the proposed attentional circuit under the conditions of our experiment (i.e., animals were highly motivated and attentive in the task, task performance was below ceiling level, A24b/M2-V1$_{axon}$ activity did fluctuate substantially), further studies with more explicit attentional manipulations may be of interest in teasing out possible contexts where this circuit does endogenously serve an attentional function.

A second limitation of our study is regarding our assertion that the optogenetic stimulation previously shown to enhance discrimination performance (Zhang et al., 2014) could be outside of the range of normal activity of A24b/M2-V1$_{axons}$. Ideally, this could be shown by examining the behavioral effects of optogenetic manipulation of A24b/M2-V1$_{axons}$ within the physiological range of activity they normally exhibit. Although this experiment would be potentially informative, there are some limitations to what could feasibly be done in this respect. For example, while ACC population activity could be optogenetically manipulated

to increase to some mean elevated target level of firing, what the target level of activity would be is not clear. Even given this manipulation, altered ACC activity would still be far from physiological in the degree of synchrony, the change in firing rate of individual cells, and the known functionally distinct subpopulations being activated in the particular context and in different phases of the trial.

A final limitation of our study is regarding the hemisphere-specific biases we observe in the activation of licking-driven A24b/M2-V1$_{axons}$. While we interpret these biases as being related to the hemisphere that is encoding the stimulus guiding behavior, it is possible that they could be due to differences in tongue and other movements between contralateral and ipsilateral stimulus trials. While this is conceivable, we consider it unlikely due to the central position of the single lick spout used in the study.

STAR★METHODS

Detailed methods are provided in the online version of this paper and include the following:

- KEY RESOURCES TABLE
- RESOURCE AVAILABILITY
  - Lead contact
  - Materials availability
  - Data and code availability
- EXPERIMENTAL MODEL AND SUBJECT DETAILS
  - Animals
- METHOD DETAILS
  - Animal surgical preparation and virus injection
  - *In vivo* imaging
  - Passive visual stimuli
  - Pupil tracking
  - Behavior
  - Calcium imaging data processing
- QUANTIFICATION AND STATISTICAL ANALYSIS
  - Analysis of activity of A24b/M2-V1$_{axons}$ and other variables during behavior
  - Analysis of activity of V1 neurons and A24b/M2-V1$_{axons}$ during passive visual stimulation
  - Other statistical methods

SUPPLEMENTAL INFORMATION

Supplemental information can be found online at https://doi.org/10.1016/j.celrep.2022.110932.

ACKNOWLEDGMENTS

This work was supported by a BBSRC-funded PhD studentship (SWBio DTP) to E.B., F.S., and A.R.; a Sêr Cymru Fellowship (80762-CU-080) to A.R.; a Wellcome Trust ISSF Seedcorn Award (105613/Z/14/Z) to A.R.; and grant PDI 2019-109285GA-I00 from the Spanish Secretary of Research, Development and Innovation (MINECO) to A.R.

AUTHOR CONTRIBUTIONS

A.R., F.S., and W.M.C. conceptualized this work. E.B., A.R., W.M.C., and V.I. analyzed the data. E.B. and A.R. performed the experiments. A.R. wrote the original draft. A.R., F.S., W.M.C., and E.B. reviewed and edited the paper. A.R., F.S., and W.M.C. assembled the team, directed the study, and secured resources and funding.

DECLARATION OF INTERESTS

The authors declare no competing interests.

Received: October 22, 2021
Revised: January 28, 2022
Accepted: May 18, 2022
Published: June 7, 2022

REFERENCES

Andermann, M.L., Kerlin, A.M., and Reid, R.C. (2010). Chronic cellular imaging of mouse visual cortex during operant behavior and passive viewing. Front. Cell. Neurosci. *4*, 3. https://doi.org/10.3389/fncel.2010.00003.

Armstrong, K.M., Fitzgerald, J.K., and Moore, T. (2006). Changes in visual receptive fields with microstimulation of frontal cortex. Neuron *50*, 791–798. https://doi.org/10.1016/j.neuron.2006.05.010.

Ayaz, A., Saleem, A.B., Schölvinck, M.L., and Carandini, M. (2013). Locomotion controls spatial integration in mouse visual cortex. Curr. Biol. *23*, 890–894. https://doi.org/10.1016/j.cub.2013.04.012.

Bahrami, B., Lavie, N., and Rees, G. (2007). Attentional load modulates responses of human primary visual cortex to invisible stimuli. Curr. Biol. *17*, 509–513. https://doi.org/10.1016/j.cub.2007.01.070.

Brainard, D.H. (1997). The psychophysics toolbox. Spat. Vis. *10*, 433–436. https://doi.org/10.1163/156856897x00357.

Broussard, G.J., Liang, Y., Fridman, M., Unger, E.K., Meng, G., Xiao, X., Ji, N., Petreanu, L., and Tian, L. (2018). In vivo measurement of afferent activity with axon-specific calcium imaging. Nat. Neurosci. *21*, 1272–1280. https://doi.org/10.1038/s41593-018-0211-4.

Bush, G., Vogt, B.A., Holmes, J., Dale, A.M., Greve, D., Jenike, M.A., and Rosen, B.R. (2002). Dorsal anterior cingulate cortex: a role in reward-based decision making. Proc. Natl. Acad. Sci. U S A *99*, 523–528. https://doi.org/10.1073/pnas.012470999.

Carandini, M., and Ferster, D. (2000). Membrane potential and firing rate in cat primary visual cortex. J Neurosci. *20*, 470–484.

Carter, C.S., Braver, T.S., Barch, D.M., Botvinick, M.M., Noll, D., and Cohen, J.D. (1998). Anterior cingulate cortex, error detection, and the online monitoring of performance. Science *280*, 747–749. https://doi.org/10.1126/science.280.5364.747.

Chen, T.-W., Wardill, T.J., Sun, Y., Pulver, S.R., Renninger, S.L., Baohan, A., Schreiter, E.R., Kerr, R.A., Orger, M.B., Jayaraman, V., et al. (2013). Ultrasensitive fluorescent proteins for imaging neuronal activity. Nature *499*, 295–300. https://doi.org/10.1038/nature12354.

Dana, H., Mohar, B., Sun, Y., Narayan, S., Gordus, A., Hasseman, J.P., Tsegaye, G., Holt, G.T., Hu, A., Walpita, D., et al. (2016). Sensitive red protein calcium indicators for imaging neural activity. Elife *5*, e12727. https://doi.org/10.7554/eLife.12727.

Fiser, A., Mahringer, D., Oyibo, H.K., Petersen, A.V., Leinweber, M., and Keller, G.B. (2016). Experience-dependent spatial expectations in mouse visual cortex. Nat. Neurosci. *19*, 1658–1664. https://doi.org/10.1038/nn.4385.

Gilbert, C.D., and Li, W. (2013). Top-down influences on visual processing. Nat. Rev. Neurosci. *14*, 350–363. https://doi.org/10.1038/nrn3476.

Goldey, G.J., Roumis, D.K., Glickfeld, L.L., Kerlin, A.M., Reid, R.C., Bonin, V., Schafer, D.P., and Andermann, M.L. (2014). Removable cranial windows for long-term imaging in awake mice. Nat. Protoc. *9*, 2515–2538. https://doi.org/10.1038/nprot.2014.165.

Huda, R., Sipe, G.O., Breton-Provencher, V., Cruz, K.G., Pho, G.N., Adam, E., Gunter, L.M., Sullins, A., Wickersham, I.R., and Sur, M. (2020). Distinct prefrontal top-down circuits differentially modulate sensorimotor behavior. Nat. Commun. *11*, 6007. https://doi.org/10.1038/s41467-020-19772-z.

Keller, A.J., Houlton, R., Kampa, B.M., Lesica, N.A., Mrsic-Flogel, T.D., Keller, G.B., and Helmchen, F. (2017). Stimulus relevance modulates contrast adaptation in visual cortex. Elife *6*, e21589. https://doi.org/10.7554/elife.21589.

Keller, G.B., Bonhoeffer, T., and Hübener, M. (2012). Sensorimotor mismatch signals in primary visual cortex of the behaving mouse. Neuron *74*, 809–815. https://doi.org/10.1016/j.neuron.2012.03.040.

Kim, J.-H., Ma, D.-H., Jung, E., Choi, I., and Lee, S.-H. (2021). Gated feedforward inhibition in the frontal cortex releases goal-directed action. Nat. Neurosci. *24*, 1452–1464. https://doi.org/10.1038/s41593-021-00910-9.

Knudsen, E.I. (2007). Fundamental components of attention. Annu. Rev. Neurosci. *30*, 57–78. https://doi.org/10.1146/annurev.neuro.30.051606.094256.

Laubach, M., Amarante, L.M., Swanson, K., and White, S.R. (2018). What, if anything, is rodent prefrontal cortex? eNeuro *5*, ENEURO.0315-18.2018. https://doi.org/10.1523/eneuro.0315-18.2018.

Leinweber, M., Ward, D.R., Sobczak, J.M., Attinger, A., and Keller, G.B. (2017). A sensorimotor circuit in mouse cortex for visual flow predictions. Neuron *96*, 1204–1432.e5. https://doi.org/10.1016/j.neuron.2017.11.009.

Makino, H., and Komiyama, T. (2015). Learning enhances the relative impact of top-down processing in the visual cortex. Nat. Neurosci. *18*, 1116–1122. https://doi.org/10.1038/nn.4061.

Mathis, A., Mamidanna, P., Cury, K.M., Abe, T., Murthy, V.N., Mathis, M.W., and Bethge, M. (2018). DeepLabCut: markerless pose estimation of user-defined body parts with deep learning. Nat. Neurosci. *21*, 1281–1289. https://doi.org/10.1038/s41593-018-0209-y.

Moore, T., and Armstrong, K.M. (2003). Selective gating of visual signals by microstimulation of frontal cortex. Nature *421*, 370–373. https://doi.org/10.1038/nature01341.

Moore, T., and Zirnsak, M. (2017). Neural mechanisms of selective visual attention. Annu. Rev. Psychol. *68*, 47–72. https://doi.org/10.1146/annurev-psych-122414-033400.

Morimoto, M.M., Uchishiba, E., and Saleem, A.B. (2021). Organization of feedback projections to mouse primary visual cortex. iScience *24*, 102450. https://doi.org/10.1016/j.isci.2021.102450.

Nath, T., Mathis, A., Chen, A.C., Patel, A., Bethge, M., and Mathis, M.W. (2019). Using DeepLabCut for 3D markerless pose estimation across species and behaviors. Nat. Protoc. *14*, 2152–2176. https://doi.org/10.1038/s41596-019-0176-0.

Niell, C.M., and Stryker, M.P. (2010). Modulation of visual responses by behavioral state in mouse visual cortex. Neuron *65*, 472–479. https://doi.org/10.1016/j.neuron.2010.01.033.

Norman, K.J., Riceberg, J.S., Koike, H., Bateh, J., McCraney, S.E., Caro, K., Kato, D., Liang, A., Yamamuro, K., Flanigan, M.E., et al. (2021a). Post-error recruitment of frontal sensory cortical projections promotes attention in mice. Neuron *109*, 1202–1213.e5. https://doi.org/10.1016/j.neuron.2021.02.001.

Norman, K.J., Bateh, J., Maccario, P., Cho, C., Caro, K., Koike, H., and Morishita, H. (2021b). Task demand-dependent contribution of frontal-sensory cortical projections to attentional behavior in mice. Preprint at bioRxiv. https://doi.org/10.1101/2021.04.08.439093.

Pachitariu, M., Stringer, C., Schröder, S., Dipoppa, M., Rossi, L.F., Carandini, M., and Harris, K.D. (2016). Suite2p: beyond 10,000 neurons with standard two-photon microscopy. bioRxiv. https://doi.org/10.1101/061507.

Pakan, J.M.P., Currie, S.P., Fischer, L., and Rochefort, N.L. (2018). The impact of visual cues, reward, and motor feedback on the representation of behaviorally relevant spatial locations in primary visual cortex. Cell Rep. *24*, 2521–2528. https://doi.org/10.1016/j.celrep.2018.08.010.

Passingham, R.E., and Wise, S.P. (2012). The Neurobiology of the Prefrontal Cortex: Anatomy, Evolution, and the Origin of Insight (Oxford University Press), pp. 88–89.

Poort, J., Khan, A.G., Pachitariu, M., Nemri, A., Orsolic, I., Krupic, J., Bauza, M., Sahani, M., Keller, G.B., Mrsic-Flogel, T.D., and Hofer, S. (2015). Learning enhances sensory and multiple non-sensory representations in primary visual cortex. Neuron *86*, 1478–1490. https://doi.org/10.1016/j.neuron.2015.05.037.

Ranson, A. (2017). Stability and plasticity of contextual modulation in the mouse visual cortex. Cell Rep. *18*, 840–848. https://doi.org/10.1016/j.celrep.2016.12.080.

Ranson, A., Sengpiel, F., and Fox, K. (2013). The role of GluA1 in ocular dominance plasticity in the mouse visual cortex. J. Neurosci. *33*, 15220–15225. https://doi.org/10.1523/jneurosci.2078-13.2013.

Reynolds, J.H., and Heeger, D.J. (2009). The normalization model of attention. Neuron *61*, 168–185. https://doi.org/10.1016/j.neuron.2009.01.002.

Saleem, A.B., Ayaz, A., Jeffery, K.J., Harris, K.D., and Carandini, M. (2013). Integration of visual motion and locomotion in mouse visual cortex. Nat. Neurosci. *16*, 1864–1869. https://doi.org/10.1038/nn.3567.

Saleem, A.B., Diamanti, E.M., Fournier, J., Harris, K.D., and Carandini, M. (2018). Coherent encoding of subjective spatial position in visual cortex and hippocampus. Nature *562*, 124–127. https://doi.org/10.1038/s41586-018-0516-1.

Schuler, M., and Bear, M. (2009). Reward timing in the PRimary visual cortex. Science *311*, 1606–1610.

Shenhav, A., Botvinick, M.M., and Cohen, J.D. (2013). The expected value of control: an integrative theory of anterior cingulate cortex function. Neuron *79*, 217–240. https://doi.org/10.1016/j.neuron.2013.07.007.

Sinnamon, H., and Galer, B. (1984). Head movements elicited by electrical stimulation of the anteromedial cortex of the rat. Physiol. Behav. *33*, 185–190. https://doi.org/10.1016/0031-9384(84)90098-2.

Speed, A., Del Rosario, J., Mikail, N., and Haider, B. (2020). Spatial attention enhances network, cellular and subthreshold responses in mouse visual cortex. Nat. Commun. *11*, 505. https://doi.org/10.1038/s41467-020-14355-4.

Stringer, C., Michaelos, M., Tsyboulski, D., Lindo, S.E., and Pachitariu, M. (2021). High-precision coding in visual cortex. Cell *184*, 2767–2778.e15. https://doi.org/10.1016/j.cell.2021.03.042.

Sundberg, K.A., Mitchell, J.F., and Reynolds, J.H. (2009). Spatial attention modulates center-surround interactions in macaque visual area V4. Neuron *61*, 952–963. https://doi.org/10.1016/j.neuron.2009.02.023.

Winkowski, D.E., and Knudsen, E.I. (2006). Top-down gain control of the auditory space map by gaze control circuitry in the barn owl. Nature *439*, 336–339. https://doi.org/10.1038/nature04411.

Zhang, S., Xu, M., Kamigaki, T., Hoang Do, J.P., Chang, W.-C., Jenvay, S., Miyamichi, K., Luo, L., and Dan, Y. (2014). Long-range and local circuits for top-down modulation of visual cortex processing. Science *345*, 660–665. https://doi.org/10.1126/science.1254126.

STAR★METHODS

KEY RESOURCES TABLE

REAGENT or RESOURCE	SOURCE	IDENTIFIER
Bacterial and virus strains		
AAV.Syn.GCaMP6s.WPRE.SV40	Addgene	100843
AAV1.Syn.NES-jRGECO1a.WPRE.SV40	Addgene	100854
AAV-hSynapsin1-axon-GCaMP6s	Addgene	111262
Chemicals, peptides, and recombinant proteins		
Sucrose	Sigma-Alderich	S0389
Cherry Kool-Aid	Kool-Aid	N/A
Enrofloxacin	Bayer	QJ01MA90
Carprofen	Zoetis	QM01AE91
Dexamethasone	MSG	50-02-2
Experimental models: Organisms/strains		
C57BL/6J mice	Jackson	000664
Software and algorithms		
MATLAB	Mathworks Inc	N/A
Python 3.8	https://www.python.org/downloads/release/python-380/	N/A
DeepLabCut	https://github.com/DeepLabCut/DeepLabCut	N/A
PsychToolbox	http://psychtoolbox.org	N/A
Suite2P	https://github.com/MouseLand/suite2p	N/A
Scanimage 4.1	http://scanimage.vidriotechnologies.com	N/A
Labjack API for interfacing with lick detector	https://labjack.com	N/A
Python code for video analysis	Zenodo	https://doi.org/10.5281/zenodo.6530962
MATLAB code for calcium signal analysis	Zenodo	https://doi.org/10.5281/zenodo.6531829
MATLAB code for intrinsic signal analysis	Zenodo	https://doi.org/10.5281/zenodo.6531804
MATLAB code for behavioral paradigm and online analysis	Zenodo	https://doi.org/10.5281/zenodo.6530995
Arduino code for capacitive lick sensor	Zenodo	https://doi.org/10.5281/zenodo.6531555
Other		
Super Bond C&B dental cement	https://www.prestige-dental.co.uk/	7112-350
3 mm circular glass	Harvard Apparatus	64-0720(CS-3R)
4 mm circular glass	Harvard Apparatus	64-0724(CS-4R)
UV curing optical adhesive	Thorlabs	7106
Vetbond	WPI	VETBOND
MAKO G-125B camera for intrinsic signal imaging	Stemmer	AVT MAKO G-125B POE
DMK 22AUC03 camera for eye imaging	Image Source	DMK 22AUC03
Labjack U3-LV data USB acquisition system	Lackjack	U3-LV
Solenoid valve for airpuff and fluid delivery	Neptune Research	161T011

RESOURCE AVAILABILITY

Lead contact

Further information and requests for resources and reagents should be directed to and will be fulfilled by the lead contact, Adam Ranson (aranson@uic.es).

Materials availability

The study did not generate new reagents.

Data and code availability

Microscopy data reported in this paper will be shared by the lead contact upon request. All original code has been deposited at Zenodo and is publicly available as of the date of publication. The DOI is listed in the key resources table. Any additional information required to reanalyze the data reported in this paper is available from the lead contact upon request.

EXPERIMENTAL MODEL AND SUBJECT DETAILS

Animals

All experimental procedures were carried out in accordance with institutional animal welfare guidelines and licensed by the UK Home Office. Experiments were carried out on adult C57BL/6J mice (aged > P90) of either sex. Numbers of animals used in each analysis are listed in the text. Mice were housed under normal light conditions (14 h light, 10 h dark) and recordings were made during the light period. Animals were given ad libitum access to food and water except during periods of behavioral training during which they were water restricted as described in behavior section. Animals were housed in transparent plastic cages with at least one other animal.

METHOD DETAILS

Animal surgical preparation and virus injection

Aseptic surgical procedures were conducted based on previously described protocols (Goldey et al., 2014; Ranson, 2017). Approximately one hour prior to cranial window surgery and virus injection, animals were administered with the antibiotic Enrofloxacin (5 mg/kg, s.c.) and the anti-inflammatory drugs Carprofen (5 mg/kg, s.c.) and Dexamethasone (0.15 mg/Kg, i.m.). Anesthesia was induced and maintained using isoflurane at concentrations of 4%, and 1.5–2% respectively. After animals were stereotaxically secured, the scalp and periosteum were removed from the dorsal surface of the skull, and a custom head plate was attached to the cranium using dental cement (Super Bond C&B), with an aperture approximately centered over right V1. A 3 mm circular craniotomy was next performed, centered on the stereotaxically identified monocular portion of V1.

For injections into V1, intrinsic signal imaging was used (after skull exposure but before craniotomy) to localise monocular V1. After intrinsic signal imaging, injections of an AAV to drive expression of jRGECO1a (AAV1.Syn.NES-jRGECO1a.WPRE.SV40; titre after dilution 5×10^{12} GC/mL; volume 100 nL) (Dana et al., 2016) were targeted using a functional map of retinotopy overlaid on surface vasculature (depth 250 μm). For injections into ACC, a small craniotomy was first made over the region (centered at 0.2–0.3 mm anterior and 0.3 lateral of bregma) either using a dental drill or by thinning the overlying bone and then piercing a small hole using a hypodermic needle. After craniotomy an AAV was injected to drive expression of GCaMP6s (AAV1.Syn.GCaMP6s.WPRE.SV40; titre after dilution 2×10^{11} GC/mL; volume 100 nL; used in behavioral experiments) (Chen et al., 2013) or axon-GCaMP6s (rAAV2/1-hSynapsin1-axon-GCaMP6s; titre after dilution 5×10^{11} GC/mL; volume 100 nL; used in passive visual stimulation experiments) (Broussard et al., 2018). All injections were made using a Nanoject II system (Drummond Scientific Company) at a rate of 10 nL/min using pulled and bevelled oil filled glass micropipettes with a tip outer diameter of approximately 30 μm. After injections the craniotomy over V1 was closed with a glass insert constructed from 3 layers of circular no 1 thickness glass (1 × 4 mm, 2 × 3 mm diameter) bonded together with UV cured optical adhesive (Norland Products; catalog no. 7106), and the craniotomy over ACC was closed with Vetbond. After surgery animals were allowed at least 2 weeks to recover after which they were either habituated to head fixation during passive visual stimulation or during visual discrimination training.

In vivo imaging

In vivo 2-photon imaging was performed using a resonant scanning microscope (Thorlabs, B-Scope) with a 16x 0.8NA objective with 3 mm working distance (Nikon) and 525/50 and 607/70 band pass emission filters. Genetically encoded calcium indicators were excited at 920–980 nm using a Ti:sapphire laser (Coherent, Chameleon) with a maximum laser power at sample of 50 mW. In single plane experiments, data was acquired at a resolution of 256 × 256 pixels at a framerate of approximately 60 Hz and averaged, resulting in a framerate of approximately 10 Hz. In multiple plane experiments, data was acquired from 6 planes (including one discarded ‘fly back’ plane) at a resolution of 256 × 256 pixels at a framerate of approximately 30 Hz, resulting in each plane being sampled at approximately 5 Hz.The field of view size was 282 × 282 μm in single plane experiments and 222 × 222 μm in multi-plane experiments. Imaging, behavioral and visual stimulation timing data were acquired using Scanimage 4.1 and custom written code (MATLAB) and a DAQ card (NI PCIe-6323, National Instruments). During imaging, animals were placed on a 20 cm diameter cylindrical treadmill which was locked in position. *In vivo* intrinsic signal imaging was performed using previously described methods using either a custom built system based around a MAKO G-125B camera (AVT) or a commercially available system (Imager 3001, Optical Imaging Inc.) (Ranson et al., 2013).

Passive visual stimuli

For recordings of passive visual responses (i.e. not during behaviour) mice were stimulated with a circular 20 × 20° drifting horizontal square wave gratings with temporal frequency of 2 Hz and spatial frequency of 0.05 cycles/°, and at one of 8 orientations. The stimuli were displayed at one of 32 positions arranged in a grid of 8 horizontal positions (spanning 80° of visual space) and 4 vertical positions (spanning 30° of visual space). Each stimulus appeared and drifted for 1 s after which the next stimulus was displayed. Visual stimuli

were generated in MATLAB using the psychophysics toolbox (Brainard, 1997) and displayed on calibrated LCD screens (Iiyama, BT481).

Pupil tracking

In a subset of experiments, video of the eye of the mouse was recorded using a USB monochrome camera (Imaging Source model DMK 22AUC03 with lens Azure-7524 mm) acquired using MATLAB image acquisition toolbox. The eye was illuminated with an infrared LED. To track the pupil and ascertain its diameter at each time point, 400 frames were manually labeled with twelve points – one at the superior, inferior, medial and lateral corners of the eye, and eight in an octagon around the pupil (Figure S4G). Then, using the DeepLabCut software package (Mathis et al., 2018; Nath et al., 2019), a resnet v1 50-based convolutional neural network was trained to predict the location of these markers for 1.3 million iterations. This network was then used to place these points on every frame of each video. When applied to novel videos from which frames were not included in the training dataset, the network nevertheless produced qualitatively good approximations of the pupil's size and activity.

While the network's placement was visually indistinguishable from human placement for the vast majority of frames, the network would always attempt to place all eight pupil markers, even if the mouse was blinking or the pupil was otherwise mostly obscured by the eyelids. To remedy this, a Python program was written to construct an eye shape by passing a pair of parabolic curves through the medial, superior, and lateral and medial, inferior, and lateral eye markers respectively, and any pupil markers that fell outside this eye shape were discarded (Figure S4G). In addition, if a set of basic assumptions about the eye shape were violated – for instance, if the medial eye marker was lateral to the lateral eye marker – all pupil markers were discarded. Finally, if six or more pupil markers were valid, an ellipse was fitted to them to minimize the least-squared error. Together these measures resulted in high fidelity tracking of pupil behavior across frames.

Behavior

Animals were trained in a go/no-go task similar to that previously described (Andermann et al., 2010; Zhang et al., 2014), and implemented using custom written code in MATLAB using the psychophysics toolbox for visual stimulus and sound generation (Brainard, 1997). In the task, animals had to lick in response to a vertically oriented nasally drifting grating for water reward (go condition) or suppress licking in response to a horizontally oriented upward drifting grating (no-go condition). Grating stimuli occupied approximately 80° of visual field and were presented with temporal frequency of 1 Hz and spatial frequency of 0.05 cycles/° in the monocular visual field. Each correct trial was rewarded with 5μL of a solution made up of 500 mL water, 50g sucrose and 1.7g Koolaid. Licking was detected using a custom-made capacitive lick sensor. Rewards were delivered using a reward valve (Neptune Research 161T011) controlled using a custom-made circuit triggered with a digital signal of calibrated duration from a data acquisition device (Labjack, U3) interfaced with using MATLAB. Incorrect no-go trials were punished with a 500 ms air puff, 500 ms white noise auditory stimulus and a ten second time-out. Several days prior to commencing training, mice were placed on water restriction and behavioral training commenced after they reached approximately 80% of their initial weight. During the initial stage of training no visual stimuli were presented to the mice and animals could obtain a reward by licking during windows of up to 60 s. If animals licked during this period they were rewarded, and this was followed by a variable duration period (2–10 s) during which licking was not rewarded. During the variable delay period (termed the quiescent period) animals had to suppress licking or the next trial did not start. This quiescent period was maintained during the entire training and testing procedure. Once mice were consistently licking the spout, they progressed to the next stage during which trials were initiated with a pure auditory tone (0.1 s, frequency of 5 kHz), followed 1 s after tone onset by 4 s of go stimulus presentation. The mouse was rewarded for licking during the 2 to 4 s period after go-stimulus onset - i.e., responses during the initial 2 s period after stimulus onset were disregarded. Disregarding licks immediately after stimulus onset was important as often animals exhibited impulsive licking at stimulus onset which was unrelated to stimulus type. During this training period, in a gradually decreasing fraction of trials (starting at 90% of trials), free rewards were administered during the go stimulus period, even if the animal did not respond. This fraction of free reward trials was automatically decreased in steps of 10% if animals responded independently and correctly in blocks of 20–30 trials. Once animals were responding correctly to 70% of go stimuli the no-go stimulus was introduced, and free reward trials were excluded. Once animals reached a go/no-go discrimination accuracy of d' > 1.5 they were classified as fully trained and at his point experimental imaging data was typically collected. The discrimination index d' was calculated as norminv(hit rate)-norminv(false alarm rate), where norminv is the inverse of the cumulative normal function, hit rate is the fraction of go trials in which animals licked, and false alarm rate is the fraction of no-go trials in which animals licked. Larger d′ values are indicative of higher visual discrimination performance. In the 'bilateral' version of the task, in each trial stimuli were presented in either the left or right monocular visual field, but the training procedures were as above. In the bilateral task, training progress was measured, and training stage advanced, using overall accuracy (i.e. pooled over left and right trial types) rather than in a side specific manner.

Calcium imaging data processing

Calcium imaging data was registered and segregated into neuronal regions of interest (i.e. A24b/M2 axons or V1 somas) using Suite2P (Pachitariu et al., 2016). The time series of each ROI was then converted from a raw fluorescence value to ΔF/F with the denominator F value trace constructed by calculating the 5th percentile of the smoothed F value within a 20 s window centered on each sample in the F trace. Boutons with correlation coefficients of >0.7 were considered to be from the same axon and combined using

a weighted average, with weighting determined by the number of pixels in the ROI of the bouton. In dual-colour imaging experiments we assessed the possibility of cross-talk between imaging channels. This could in particular be a problem when imaging boutons in layer 1 of the visual cortex (visualised on the green channel; GCaMP6-axon) because they are expected to be surrounded by a larger volume of dendrites and axons of labeled V1 cells (visualised on the red channel; jRGECO1a). We therefore assessed the extent to which dendrite and axon originating red signals might be bleeding into the green channel. To quantify this issue in our experiments, for each bouton ROI we regressed fluorescence from the green channel against fluorescence from the red channel (i.e. for the same pixels) which provided us with a slope of the relationship between these variables, as well as an R^2 value (i.e. the fraction of variance of the fluorescence of the green channel of the ROI which could be explained by the fluorescence of the red channel of the ROI). We found that while 77% of boutons had a positive slope (50% would be the chance level, suggesting a degree of bleed through or some other shared source of variation), the average R^2 value was 0.0059. That is to say, less than 1% of the variance of the green channel signal could be explained by the red channel signal. For this reason, we consider the issue of bleed through to be negligible in our experiments.

QUANTIFICATION AND STATISTICAL ANALYSIS

Analysis of activity of A24b/M2-V1$_{axons}$ and other variables during behavior

Whether boutons were overall positively or negatively modulated by the task (Figures 1C and 1D) was calculated by comparing within task periods (average ΔF/F value over the period from trial onset tone to the stimulus offset) to between task periods (average ΔF/F value of the final 2 s of the properly executed quiescent period immediately before trial onset; thus ensuring a period with no licking behavior). The task modulation index (Figure S2A) was calculated from these same periods with the formula (within task activity - between task activity)/(within task activity + between task activity) with an index of 1 indicating boutons are only active inside of the task and an index of −1 indicating boutons are only active during the intertrial quiescent period. Responses of boutons to individual task events (Figure 1E; tone, go stimulus, no-go stimulus, reward and airpuff/white noise) were calculated using a one-sided paired sample t-test comparison of mean ΔF/F in the 0.5–0 s before and 0–1 s after event onset, with p values reported after false discovery rate correction using the Benjamini Hochberg method.

To assess the association between the activity of individual A24b/M2-V1$_{axons}$ and task accuracy (Figures 2C, 2D, S2B, and S2C) we constructed a linear model to predict individual bouton activity in each trial, using trial correctness, trial type (go or no-go) and an interaction term as predictors. Bouton activity was calculated in each trial as the average ΔF/F value over the period from trial onset tone to the stimulus offset. The linear model was implemented using the fitglm function in MATLAB with p values corrected for multiple hypothesis testing (i.e. testing multiple axons) with FDR correction using the Benjamini Hochberg method. The correct modulation index (Figure 2C inset) was calculated as ($R_{correct}$ – $R_{incorrect}$)/($R_{correct}$ + $R_{incorrect}$) where $R_{correct}$ and $R_{incorrect}$ are responses on correct and incorrect trials respectively.

In supplemental analysis of the relationship between A24b/M2-V1$_{axon}$ activity and discrimination performance (presented in Figures S2E and S2F) we assessed the relationship between d' and A24b/M2-V1$_{axon}$ activity. To do this we first calculated d' and average A24b/M2-V1$_{axon}$ population ΔF/F over a sliding window of 20 trials (experiments typically consisted of ~200 trials) - with A24b/M2-V1$_{axon}$ population ΔF/F calculated either 1) during a 1 s period before trial onset), 2) during the initial 2 s stimulus period (during which responses don't affect trial outcome), or 3) during the response period. A straight line was then fit to the pairs of d'/A24b/M2-V1$_{axon}$ population ΔF/F data points with the best fit assessed with least squares. Each of the gray lines in Figures S2E and S2F is a fit line from one experiment, and the black line is the average of these fit lines. Statistical significance of the relationship between d' and A24b/M2-V1$_{axon}$ population ΔF/F was tested by computing whether the average slope coefficients of the fitted lines differed significantly from zero using a two-sided one sample t-test.

To measure the correlation between licking and A24b/M2-V1$_{axon}$ activity the lick raster was first converted to a lick rate by summing the licks in a 1 s window which was slid over the binary lick event trace. The correlation coefficient between lick rate and the ΔF/F of each A24b/M2-V1$_{axon}$ ROI was then calculated. Because of the non-independence of neighboring samples in the two traces we used a permutation test to test significance by building a null distribution to which the observed correlation coefficients could be compared. To do this we randomly circularly shifted the lick rate trace 1000 times, with a minimum shift of the equivalent of ±10 s, and in each instance measured the correlation coefficient between the shifted lick rate trace and ΔF/F of each A24b/M2-V1$_{axon}$ ROI. A24b/M2-V1$_{axons}$ were deemed to be significantly correlated if their observed lick rate correlation coefficient exceeded 95% of those observed in the null distribution. The comparison of rewarded vs. unrewarded licking (Figures 3B and 4E) was made by averaging ΔF/F during rewarded and unrewarded periods of licking within go trials. In analysis where we sought to control for licking frequency (Figure S4B), bouts of licking were identified using the raw lick events, which were isolated in time (preceded by at least 1 s of non-licking), and in which 3 licks occurred in the first 0.5 s of the bout. Only the neural activity during the first 3 licks in a bout were analyzed to avoid confounds associated with rewarded licking bouts differing in length from unrewarded licks. Analysis of the rate of rewarded and unrewarded licking (Figure S4D) was carried out within go trials during the unrewarded or rewarded periods. Analysis of eye movement velocity during rewarded and unrewarded periods (Figures S4H and S4I) was also carried out within go trials during the unrewarded or rewarded periods, and additionally during non-licking inter-trial periods. In order to analyze the hemispheric targeting of lick/reward signals (Figure 4F)

a targeting index was derived from the mean ΔF/F during rewarded licking in contralateral trials (C_{reward}) and ipsilateral trials (I_{reward}) using the formula:

$$(C_{reward} - I_{reward})/(C_{reward} + I_{reward})$$

Analysis of activity of V1 neurons and A24b/M2-V1$_{axons}$ during passive visual stimulation

Orientation tuning data (Figures 2G, 2H, and S3A, S3B, and S3C) were fitted using the MATLAB function lsqcurvefit and modeled as a sum of two Gaussians which were constrained such that one peaked at the preferred orientation, and the peaks were 180° apart (Carandini and Ferster, 2000), with the preferred stimulus defined as the stimulus orientation which elicited the largest response on average. The response of each neuron was the average of ΔF/F over the 1 s after stimulus onset. The orientation selectivity index (OSI) was computed from the orientation tuning fit as:

$$OSI = (R_{pref} - R_{ortho})/(R_{pref} + R_{ortho})$$

where R_{pref} and R_{ortho} are responses at the preferred and orthogonal orientation respectively. The direction selectivity index was also computed from the orientation tuning fit as:

$$DSI = \left(R_{pref\_dir} - R_{pref\_dir+180^\circ}\right)/\left(R_{pref\_dir} + R_{pref\_dir+180^\circ}\right)$$

Where R_{pref_dir} and $R_{pref\_dir+180^\circ}$ are responses at the preferred direction and preferred direction +180° respectively. The level of activity of the A24b/M2-V1$_{axon}$ population was taken as a mean of all A24b/M2-V1$_{axon}$ ROIs detected over the same period that V1 soma activity was analyzed.

Other statistical methods

Statistical analysis was carried out in MATLAB 2019b using the Statistics toolbox, and group average values are presented throughout as mean ± standard error of the mean unless otherwise noted. The statistical significance of comparisons between groups was determined using a two-sided t test or ANOVA unless otherwise noted, and p values <0.05 were considered significant. Similarity of variance and normal distribution were checked with the vartestn MATLAB function. Correction of p values for multiple comparisons were calculated using the MATLAB function multcompare using the Tukey–Kramer method unless otherwise noted. Precise group sizes were not decided in advance, but approximate group sizes were based on typical sizes used in this field in similar experiments.

Cell Reports, Volume *39*

Supplemental information

Recruitment of frontal sensory circuits during visual discrimination

Eluned Broom, Vivian Imbriotis, Frank Sengpiel, William M. Connelly, and Adam Ranson

Supplemental information

Supplemental Figures

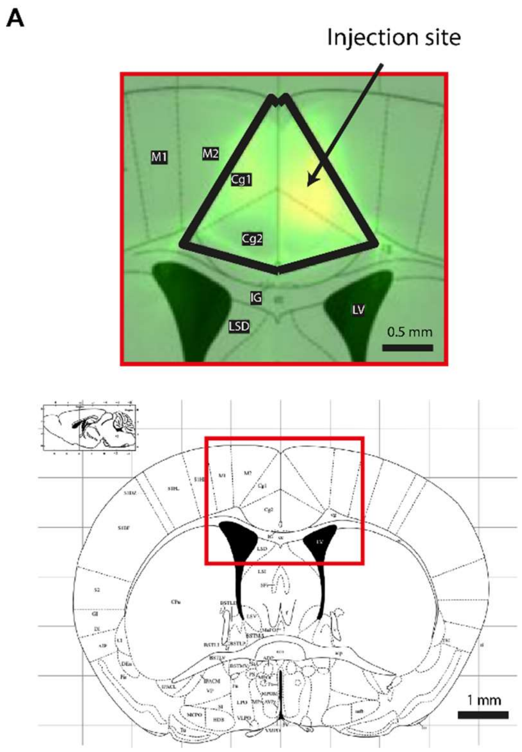

Supplemental Figure 1. The site of injection of AAV to label A24b/M2$_{axons}$. Related to Figure 1. (A) Histology from A24b injection site (labelled Cg1 in Paxinos and Franklin's the Mouse Brain in Stereotaxic Coordinates). Note, injection was unilateral, but A24b/M2 axons can be seen to project to contralateral A24b/M2.

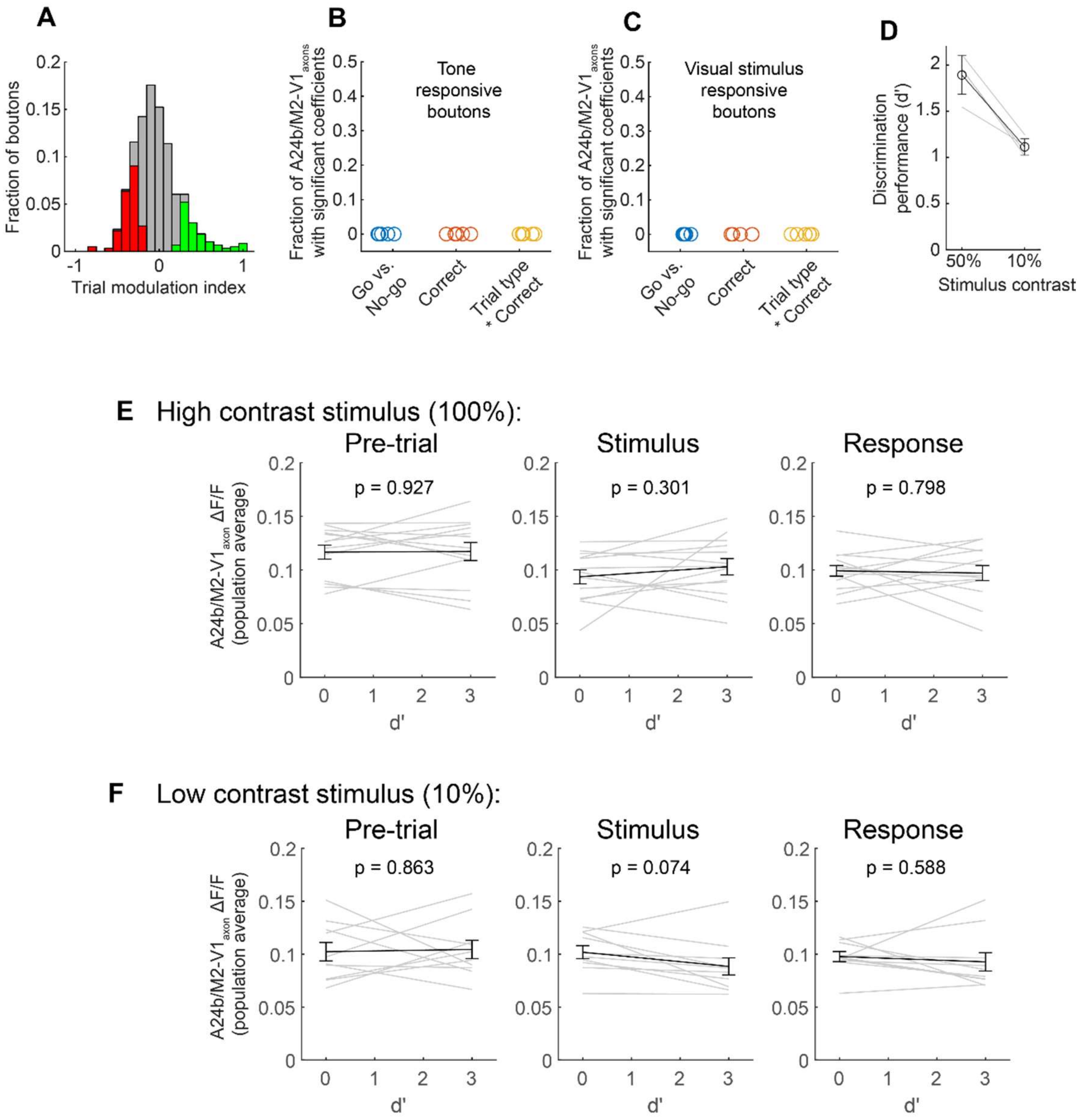

Supplemental Figure 2. Behavioural accuracy is not enhanced by increased A24b/M2-V1$_{axon}$ activity. Related to Figure 1 and 2. (A) The trial modulation index of individual A24b/M2-V1$_{axons}$ showing that there are boutons that are both positively and negatively modulated by whether the animal is within vs. between trials. 597 boutons from 5 behavioural sessions from 5 mice. (B) Behavioural accuracy (correctness) is not associated with level of activity of A24b/M2-V1$_{axons}$ (i.e. $\Delta F/F$) in tone responsive boutons. (C) As in (B) but for visually responsive boutons. (D) Discrimination accuracy reduces at lower compared to higher stimulus contrasts. (E) We observe no relationship between A24b/M2-V1axon $\Delta F/F$ and discrimination performance during the task (d'), during either the pre-trial, stimulus period or response period. Grey lines represent linear regression fits to A24b/M2-V1axon $\Delta F/F$ vs. d' in individual experiments while the black lines represent the average of these fits. P values indicate whether the slope of the fits deviate significantly from zero, as would be the case if there was a relationship between these variables. (F) As in (B) but at lower contrast (10%).

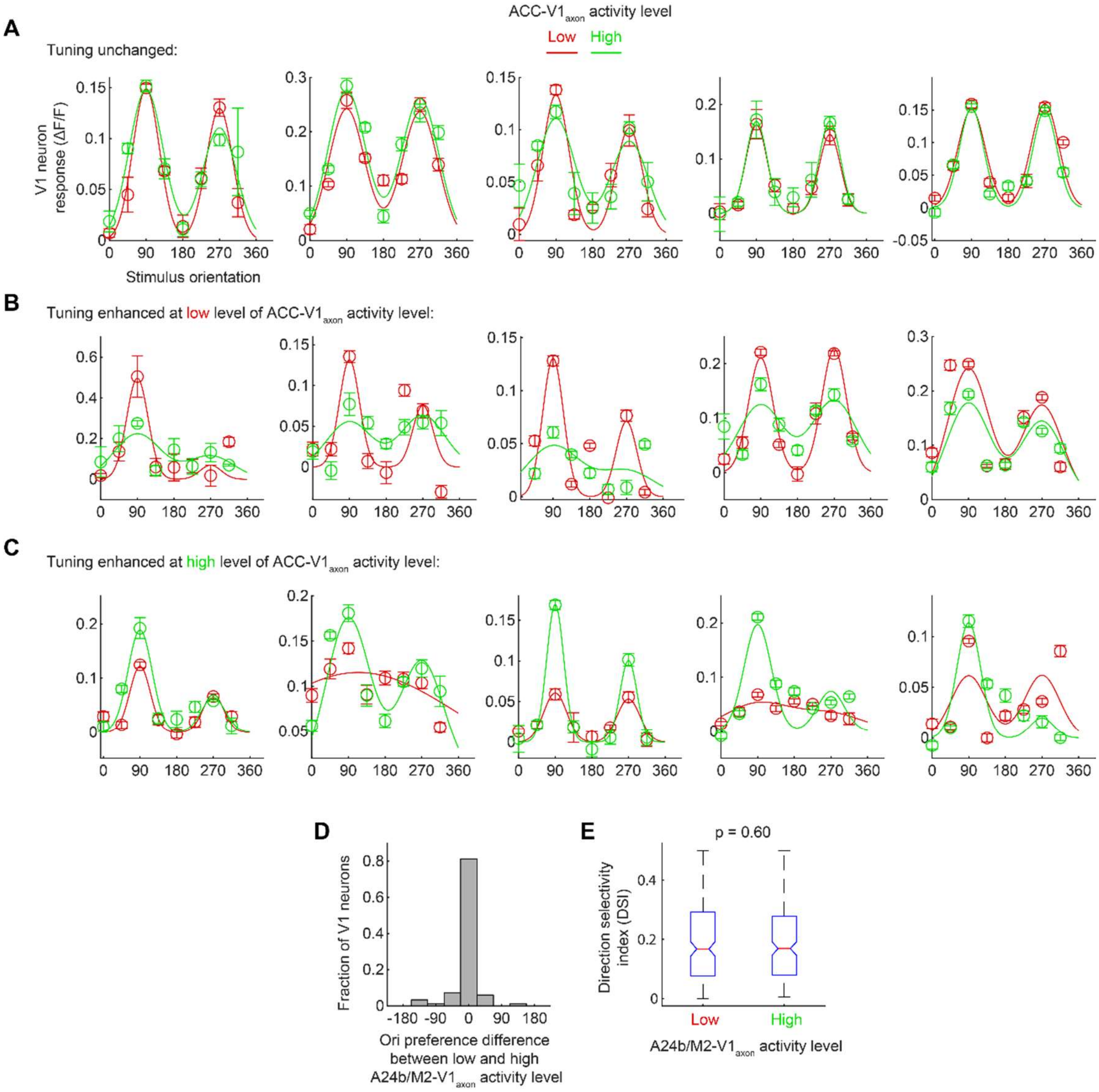

Supplemental Figure 3. Neural sensory discrimination is not enhanced by increased A24b/M2-$V1_{axon}$ activity. Related to Figure 2. (A-C) Examples of orientation tuning curves of individual V1 neurons at high (green) or low (red) levels of activity of A24b/M2-$V1_{axons}$ for neurons in which orientation selectivity was unchanged (A), decreased (B) or increased (C) at higher vs. lower levels of A24b/M2-$V1_{axon}$ activity. (D) Orientation tuning preference did not generally change at higher vs. lower levels of A24b/M2-$V1_{axon}$ activity. (E) Direction selectivity does not on average differ significantly between trials with low compared to high levels of A24b/M2-V1axon activity. Comparison of DSI in (E) was made using a Kruskal–Wallis test (n = 192 cells, from 11 experiments from 3 mice). Analysis of directional tuning was limited to cells which were classified as visually responsive (one-way ANOVA over all stimulus conditions), and for which the R^2 of orientation tuning curve fits (at both low and high levels of A24b/M2-V1axon activity) was > 0.3.

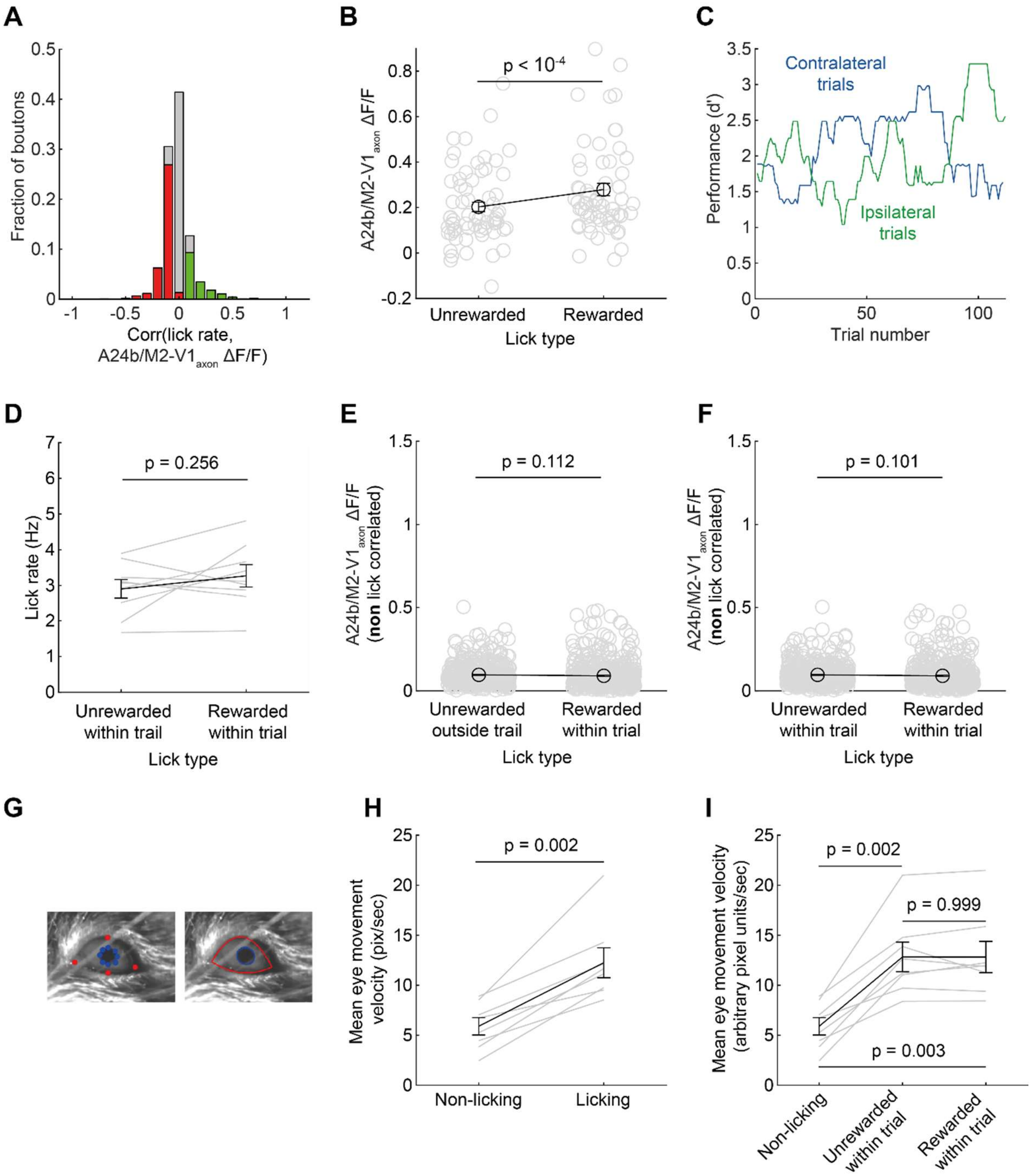

Supplemental Figure 4. A subset of A24b/M2-V1$_{axons}$ show lick correlated activity which is modulated by reward. Related to Figure 3. (A) Distribution of lick correlations of A24b/M2-V1$_{axons}$. 597 A24b/M2-V1axons, from 5 behavioural sessions from 5 mice. (B) Rewarded licks are associated with greater activity in A24b/M2-V1$_{axon}$ activity than unrewarded licks after licking pattern is controlled for. See main text and methods for description of methods used to match patterns of licking. n = 64 boutons from 5 experiments from 5 mice. (C) In some experiments accuracy on ipsilateral vs. contralateral trials varies independently suggesting hemisphere specific variations. (D) The difference between lick rate in the rewarded vs. unrewarded period of the trial was not statistically significantly different (paired sample t-test). (E) In non-lick correlated A24b/M2-V1$_{axons}$ no significant difference was observed between activity during unrewarded licking in the inter-trial period and rewarded licking during go trials. (F) As in (E) except unrewarded licking is analysed during the first 2 seconds of trials during which licking was not rewarded. (G) Left image show one frame of eye video with features detected during DeepLabCut labelled in red (eye corners and upper and lower eyelid) and blue (8 points defining the pupil). Right image shows fits to these

features to define eyelid margins (red; fit with two parabolas) and pupil (blue; ellipse). (H) Mean eye movement velocity was greater during licking as compared to non-licking periods (tested with paired sample two-sided t-test). (I) Mean eye movement velocity was similar between rewarded and unrewarded within trial periods. n = 8 experiments from 7 mice.

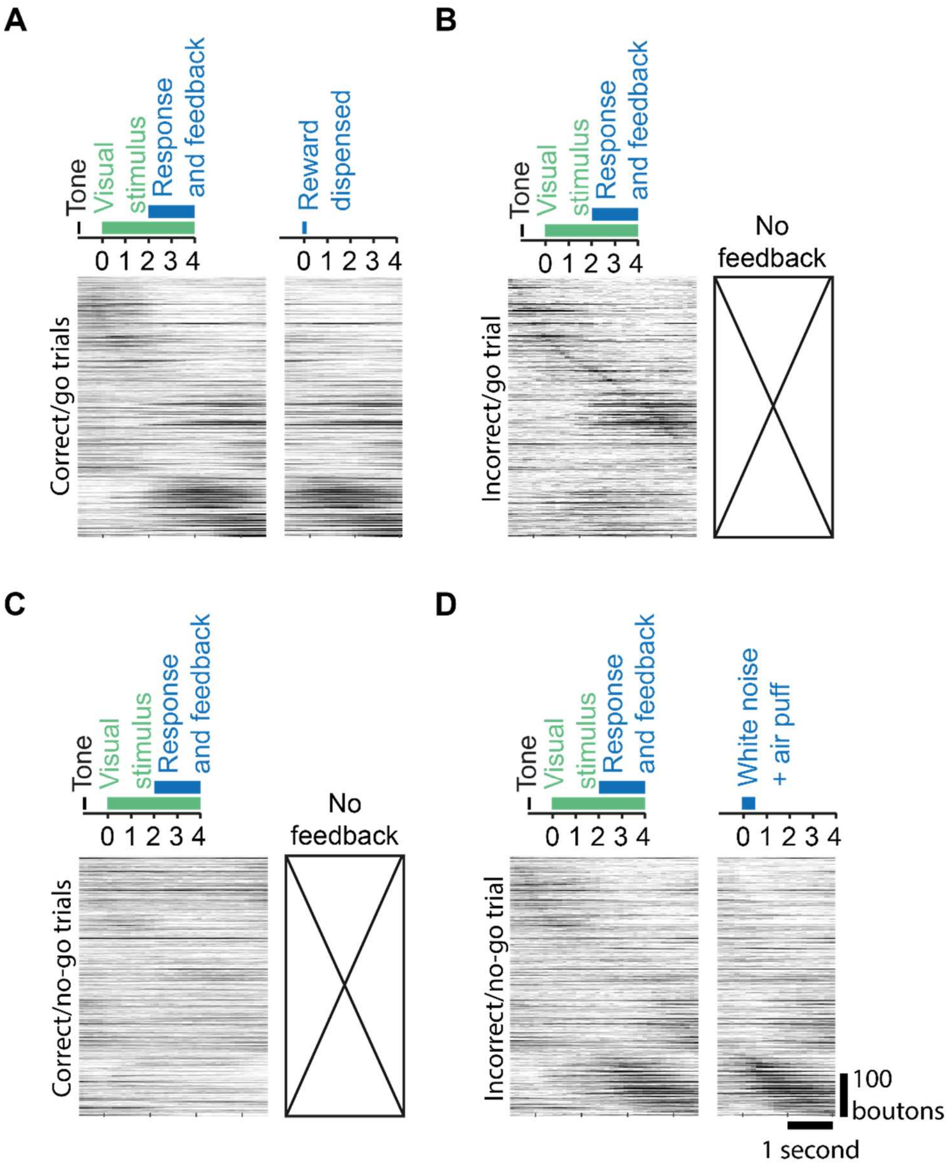

Supplemental Figure 5. Average responses of all A24b/M2-V1$_{axons}$ to all trial events. Related to Figure 1 and 2. (A) Average response of all A24b/M2-V1$_{axons}$ to all trial events in correct/go trials. Each row is a single axon. The left panel show responses to event types common to all trial types, and the right panel shows trial type specific events where appropriate (i.e. feedback). Rows are sorted by the time of maximum mean activity across all 4 trial types. (B-D) As in (A) except for incorrect/go (B), correct/no-go (C) and incorrect /no-go (D) trial types. n = 597 boutons from 5 behavioural sessions from 5 mice.

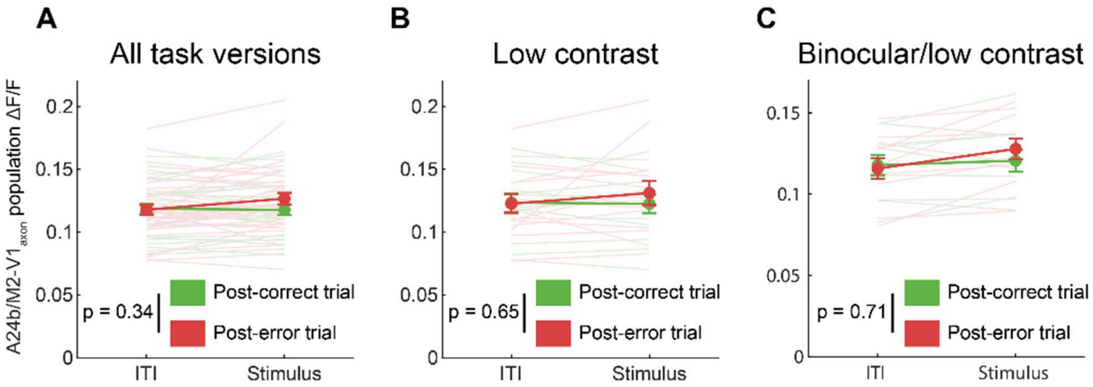

Supplemental Figure 6. Post-error recruitment of A24b/M2-V1$_{axons}$ is not observed under the conditions of our study. Related to Figure 1 and 2. A recent study reported V1 projecting cingulate neurons are selectively activated at a population level following errors during a freely moving 5-choice serial reaction time task (Norman et al., 2021a). We thus tested for activation following errors in our dataset. (A) Comparison of mean ΔF/F of boutons in each experiment post-error or post-correct trial, either during the intertrial period (ITI) or during the stimulus period of the trial. All task versions are included in analysis. Two-way ANOVA test of the effect of previous trial correctness: $p = 0.34$. $n = 32$ experiments from 11 mice. (B) As in (A) for low contrast experiments which are of higher difficulty (accuracy is reduced). Two-way ANOVA test of the effect of previous trial correctness: $p = 0.65$. $n = 13$ experiments from 7 mice. (C) As in (A) but for bilateral low contrast experiments. In this condition animals do not know in advance where the stimulus will appear, and it is of low contrast, and this is therefore arguably the most difficult version of the task. Two-way ANOVA test of the effect of previous trial correctness: $p = 0.71$. $n = 10$ experiments from 5 mice.
